# Discrimination training affects stimulus generalization in mice during Pavlovian eyeblink conditioning

**DOI:** 10.3389/fnbeh.2024.1446991

**Published:** 2024-08-23

**Authors:** Francesca Romana Fiocchi, Nikki E. S. van Dorp, Stephanie Dijkhuizen, Maurits van den Berg, Aaron Wong, Chris I. De Zeeuw, Henk-Jan Boele

**Affiliations:** ^1^Department of Neuroscience, Erasmus MC Rotterdam, Rotterdam, Netherlands; ^2^Department of Psychiatry, Washington University in St. Louis, Saint Louis, MO, United States; ^3^Princeton Neuroscience Institute, Princeton University, Princeton, NJ, United States; ^4^Royal Dutch Academy of Arts & Science (KNAW), Netherland Institute for Neuroscience, Amsterdam, Netherlands

**Keywords:** cerebellum, learning, differential training, generalization, classical conditioning

## Abstract

The delicate balance between discrimination and generalization of responses is crucial for survival in our ever-changing environment. In particular, it is important to understand how stimulus discrimination affects the level of stimulus generalization. For example, when we use non-differential training for Pavlovian eyeblink conditioning to investigate generalization of cerebellar-related eyelid motor responses, we find generalization effects on amount, amplitude and timing of the conditioned responses. However, it is unknown what the generalization effects are following differential training. We trained mice to close their eyelids to a 10 kHz tone with an air-puff as the reinforcing stimulus (CS+), while alternatingly exposing them to a tone frequency of either 4 kHz, 9 kHz or 9.5 kHz without the air-puff (CS−) during the training blocks. We tested the generalization effects during the expression of the responses after the training period with tones ranging from 2 kHz to 20 kHz. Our results show that the level of generalization tended to positively correlate with the difference between the CS+ and the CS− training stimuli. These effects of generalization were found for the probability, amplitude but not for the timing of the conditioned eyelid responses. These data indicate the specificity of the generalization effects following differential versus non-differential training, highlighting the relevance of discrimination learning for stimulus generalization.

## Introduction

In our ever-changing environment our chances for survival are enhanced by our ability to discriminate particular signals that require specific responses, but at the same time we need to be able to generalize responses across stimuli and situations that are not identical, yet require the same response. So how do we interpret different inputs? When do we emphasize the differences and when do we highlight the similarities? This delicate balance between discrimination and generalization is crucial to shape learning and optimize responses to changes in the environment. Likewise, dependent on the context the very same stimulus can evoke a discriminated response in one particular situation, but a generalized response in the other. Therefore, it is important to understand how we learn to interpret the context and how acquired discrimination of different stimuli affects generalization of responses.

Eyeblink conditioning is a form of classical conditioning that can be used to study how discrimination shapes generalization of conditioned eyelid responses. The neural substrate underlying this conditioned eyelid response has been extensively investigated (Mccormick et al., 1981; McCormick and Thompson, 1984; Jirenhed and Hesslow, 2016; Johansson et al., 2018). Purkinje cells in the cerebellar cortex play a major role in orchestrating the conditioned motor response (Boele, 2010; Schonewille et al., 2010; ten Brinke et al., 2015; Boele et al., 2016, 2018; Grasselli et al., 2020). The precise control and timing of eyelid responses during eyeblink conditioning makes it an excellent and versatile model to study learning and memory formation. Stimulus discrimination with eyeblink conditioning is established using either one of two training procedures, differential or non-differential training (Moore, 1964; Hupka et al., 1969; Liu, 1971; Moore and Mis, 1973). In differential training, animals learn to respond to a particular stimulus that is reinforced (positive conditioned stimulus, CS+), but not to a second stimulus that is not reinforced (negative conditioned stimulus, CS−). In the case of eyeblink conditioning, the CS+ is reinforced using an air-puff to the eye as an unconditioned stimulus (US), while the CS− usually has the same nature of the CS+ but with slightly different characteristics (and also does not come with the air-puff reinforcement). Instead, during non-differential training only one reinforced conditioned stimulus is used (CS+). After either differential or non-differential training, generalization is investigated by testing eyelid response recordings to different CSs, which include both the CS+ and the CS− (Ghirlanda and Enquist, 2003).

Previous studies investigated the effect of differential and non-differential procedures on eyelid conditioned response (CR) probability, revealing that the level of conditioned responses (CRs) in rabbits after differential training shows less generalization compared to non-differential training (Moore, 1964; Liu, 1971). For non-differential training, when CS stimuli are used for Pavlovian eyeblink conditioning to investigate generalization of cerebellar-related eyelid motor responses, generalization affects both amplitude and timing of the conditioned responses, in addition to the probability of responses (Fiocchi et al., 2022).

However, for differential training, it is still unknown how the similarities between CS+ and CS− during discrimination training can shape generalization of motor eyelid responses, since previous literature focused on the level of generalization following non-differential training. In the present study, we analyzed the generalization of conditioned eyelid responses following differential training using Pavlovian eyeblink conditioning in three different groups of mice. During differential training, for all groups we used a tone frequency of 10 kHz (positive conditioned stimulus, CS+) repeatedly paired with a mild air-puff to the eye (i.e., US) as the reinforced stimulus. The non-reinforced stimulus consisted of another tone frequency that was never paired with an air-puff (negative conditioned stimulus, CS−). In principle, animals can discriminate between frequencies that are as close as 4–7% apart (e.g., 6.67 kHz and 6.80 kHz as used in de Hoz and Nelken, 2014). For this reason, the tonal difference between the 10 kHz CS+ and the CS− was different for each group of animals. We decided to test CS− frequencies from a minimum of 5% (i.e., CS− of 9.5 kHz), resulting in a value that was close to the minimal threshold that mice can discriminate, to an intermediate of 10% (i.e., CS− of 9 kHz), resulting in a value above discrimination threshold, up to a maximum difference of 60% (i.e., CS− of 4 kHz).

After differential training, we tested generalization of conditioned eyelid CRs to novel tone frequencies, ranging from 2 to 20 kHz, which were never reinforced with an aversive air-puff US. We hypothesized that mice exposed to a larger tonal difference between CS+ and CS− would generalize less than animals in groups exposed to more similar frequencies during differential training. Our results show that mice differentially trained with a bigger tonal difference between CS+ and CS− show a lower generalization gradient in both the amplitude and likelihood of their eyelid responses compared to mice subjected to training conditions with a similar CS+ and CS−.

## Methods

### Subjects

We used wild-type mice B6CBAF1/JRj (*n* = 24, 10 males and 14 females) between 11 and 16 weeks old at the start of the experiment. Mice were individually housed, received food *ad libitum* and were subjected to a 12 h:12 h light/dark cycle. Experiments were performed during the light phase, and each animal was considered to be an experimental unit. Animals randomly divided in groups based on the tonal difference between CS− and CS+ (from 5 to 60% tonal difference) used during training. Our animals were divided as follows: Grp.10CS + 4CS− was given a CS− of 4 kHz (60% tonal difference, *n* = 8 of which 3 males and 5 females), 9 kHz tone for Grp.10CS + 9CS− (10% tonal difference, *n* = 8 of which 4 males and 4 females), or 9.5 kHz for Grp.10CS + 9.5CS− (5% tonal difference, *n* = 8 of which 3 males and 5 females, where 2 females were excluded from the stimulus generalization test analysis because they died during the training sessions for this reason the plots for Grp10CS + 9.5CS− indicated *n* = 6). Groups were age and sex matched. We calculated the sample size to be at least 8 animals per group. Based on the normalized eyelid closure during eyeblink conditioning, the variation between groups is substantial with a standard deviation of 0.2, which reflects about 1 SD based on previous experiments. Additionally, for the sample size calculation the power was set at 80% and a two-sided alpha at 0.05. All experiments were approved by the European Communities Council Directive for animal experiments and were in accordance with the Institutional (Erasmus MC) Animal Care and Use Committee guidelines. The Erasmus Laboratory Science center has the authority to review and approve animal experimental protocols within The Netherlands, in the same way as the Institutional Animal Care and Use Committees (IACUCs) does in the United States. Our experimental protocols were reviewed and approved by the Erasmus Laboratory Animal Science Center (work protocol nr. 15–273-137; project license nr. AVD101002015273). Experimenters were blind to tonal difference group during all experiments and primary data analysis, but not during statistical analysis. Reporting of this study is in accordance with the ARRIVE guidelines.

### Auditory brainstem responses

The B6CBAF1/JRj animals we used in our experiments result from a cross of C57BL/6 J females and CBA/J males. Previous studies show that the CBA/J mice strain has very good hearing and has been used as a reference for normal hearing in rodents (Zheng et al., 1999). Since this CBA strain is crossed with C57BL/6, which is sensitive to hearing problems with aging, we tested auditory brainstem response (ABR) of our animals before the start of the eyeblink conditioning experiments to check hearing level thresholds. Mice were anesthetized with a ketamine (Alfasan, Woerden, NL) / xylazine (Sedazine®, AST Farma, Oudewater, NL) mixture (100/10 mg/kg body weight, administered intraperitoneal) and placed in a sound- and light-attenuated box with the ears 4 cm distance from a frontally placed loudspeaker. Needle electrodes were positioned subdermal at the base of both pinnae, the external part of the ear. The reference electrode was placed at the vertex, the upper surface of the head, and a ground electrode on the lower back. Presentation of stimuli and averaging of responses were controlled by BioSigRZ software. Stimuli were generated and presented by a RZ6 Multi I/O Processor (TuckerDavis Technologies) and recorded using Medusa DA4PA Preamp. In order to determine ABR mean traces, we excluded responses above 30 μV, as these were considered artifacts. Hearing level thresholds were measured at 4, 8, 16 and 32 kHz (Willott, 2006). Thresholds were defined as the lowest sound pressure level (SPL) at which a reproducible peak was still present. After the recordings, mice were injected with atipamezole (Antisedan®, Orion Pharam, Finland) (10 mg/kg body weight, administered intraperitoneal) for the reversal of xylazine. After ABR recordings, mice had 2 days to recovery before they underwent pedestal surgery.

### Surgery

Mice were anesthetized with 2% isoflurane (vaporizer for Isoflurane Anesthetic Model100 Vaporizer, Forane®, Surgivet) and body temperature was kept constant at ~37 degrees Celsius (DC Temperature controller, FHC). After fixation in a standard mouse stereotaxic alignment system (Stoelting) and under sterile conditions, the scalp was incised (~10 mm) to expose the skull. Membranous tissue was cleared and the bone was prepared with Optibond™ FL (All-in-one bonding agent Kerr®, Salerno, Italy). A small brass pedestal with a squared magnet on top was attached to the skull with dental cement Charisma (Mitsui Chemical Group, Kulzer, Germany) using an *x*-*y*-*z* manipulator. This ensures for correct head fixation during experiments. Right after surgery, mice were allowed to recover under a heating lamp for at least 20 min until they were fully awake. They were given post-operative analgesic (Rimadyl® Cattle, Cappelle a/d IJsel, NL) on the following day and another extra day for recovery.

### Eyeblink conditioning - apparatus

**Figure 1:**
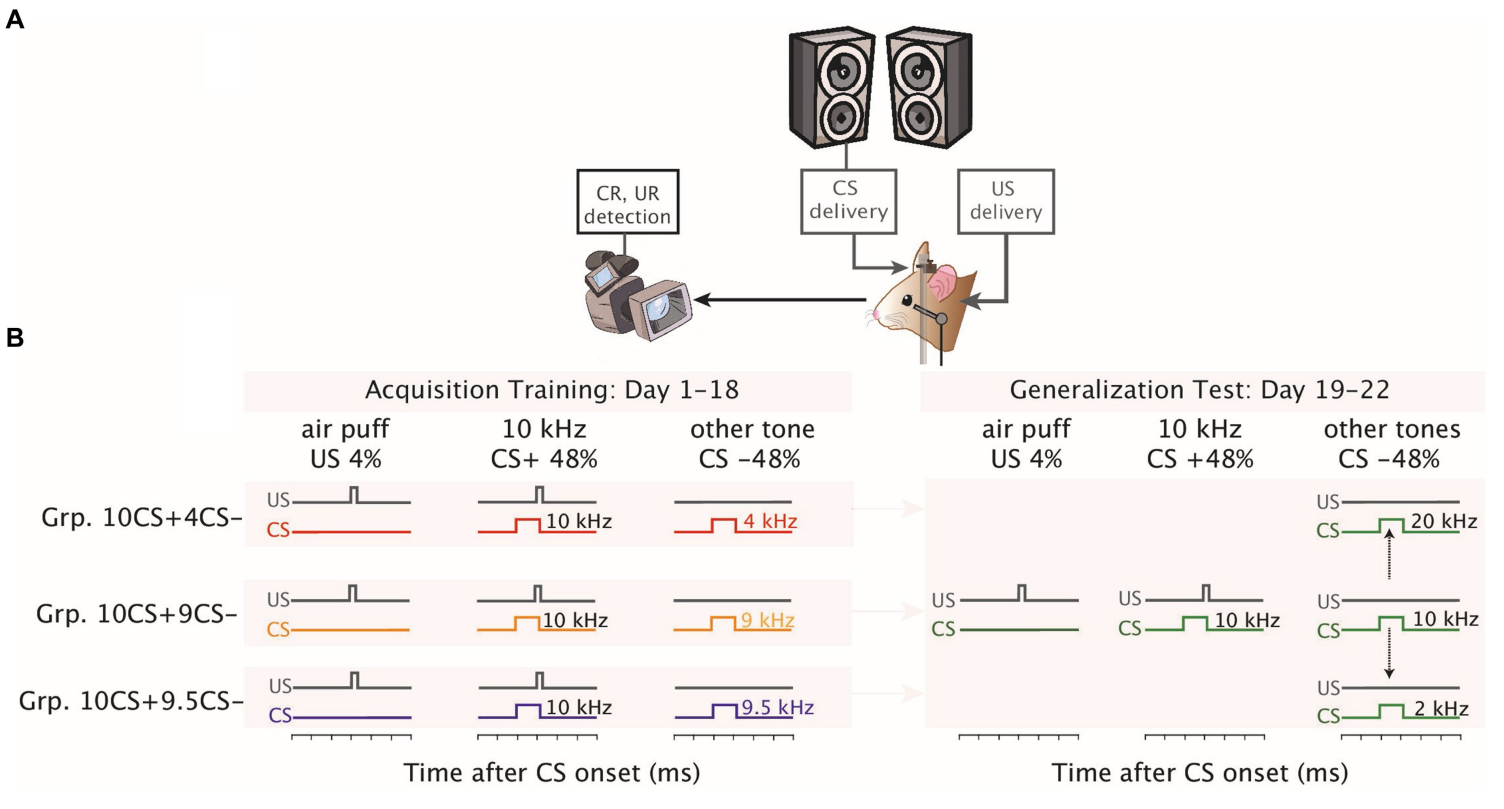
Eyeblink conditioning setup and stimulus generalization test following differential training. **(A)** Mice were placed in a light- and sound- isolating chamber on a foam cylindrical treadmill that allowed them to walk freely with their heads fixed at a horizontal bar. The conditioned stimulus (CS+) consisted of a 10 kHz tone delivered using speakers placed on both upper front corners of the chamber and the unconditioned stimulus (US) was a weak air-puff to the left eye. Eyelid movements were recorded with a high-speed video camera. Speakers were placed on both upper front corners of the chamber at the same height. Eyelid movements were recorded using a high-speed video camera system (300 frames per second). **(B)** Schematics of eyelid conditioning differential training and generalization test. For each group, on the *x*-axis the time in milliseconds illustrates the presentation of the auditory stimuli. All groups underwent the same differential training of eyeblink CRs for 1–18 consecutive days using a 10 kHz as reinforced (CS+), while the non-reinforced frequency (CS−) was a tone frequency of 4 kHz for the Grp10CS + 4CS− (in red), 9 kHz for the Grp10CS + 9CS− (in yellow) and 9.5 kHz for the Grp10CS + 9.5CS− (in blue). Afterwards, all groups were tested for 1–4 consecutive days with frequencies which were never reinforced for any of the groups. For each protocol, the duration and the ratio of different trial types is presented at the top of the corresponding illustration.

All behavioral experiments were conducted in sound- and light- attenuating boxes. Mice were placed head-fixed on top of a cylindrical treadmill on which they were allowed to walk freely (similar to Heiney et al., 2014; Boele et al., 2018). The treadmill consisted of a foam roller (diameter, ±15 cm; width, +/− 12 cm; Exervo, TeraNova EVA) with a horizontal metal rod through the axis that was connected with a ball bearing construction to two solid vertical metal poles. A horizontal messing bar was fixated to the same vertical poles at 3 to 5 cm above the treadmill. Mice were head-fixed to the bar with the use of a screw, allowing the magnet on top of the pedestal to perfectly dovetail another magnet with opposite polarity in the middle of the horizontal messing bar in the exact point of fixation, thereby ensuring perfect head stability (Figure 1A) (Chettih et al., 2011; Heiney et al., 2014; Boele et al., 2018). The CS+ for all groups was a 280 ms tone with a frequency of 10 kHz, while the CS− was a 4 kHz tone for Grp10CS + 4CS−, 9 kHz tone for Grp10CS + 9CS− and a 9.5 kHz tone for Grp10CS + 9.5CS− all with the same duration of 280 ms. Eyeblink CRs are perfectly timed responses of which the peak seems to be relatively constant during training (Boele et al., 2016). Mice trained with different ISIs are able to adjust the eyelid movements accordingly (Chettih et al., 2011). Previous experiments from our laboratory (unpublished data from Boele et al. – available upon request) show that an ISI of 280 ms represents a suitable time interval for reliable learning of eyeblink conditioning, while extending this time interval (from 600 to 800 ms) results in reduced amplitude and later peak latency of the CR. The US consisted of a 30 ms mild corneal air-puff, which was controlled by a VHS P/P solenoid valve (Lohm rate, 4,750 Lohms; Internal volume, 30 μL, The Lee Company®, Westbrook, US) and delivered via a 27.5 mm gage needle that was perpendicularly positioned at about 5-mm from the center of the left cornea. The back pressure on the solenoid valve was set at 30 psi. We used an interstimulus interval of 250 ms and an intertrial interval of 8–12 s. Eyelid movements were recorded using a high-speed video camera (333 fps, Basler® a cA640-750u m ID: 106748–15, Germany). Stimulus control and data acquisition were done with National Instruments hardware. All experiments were performed at approximately the same time of day by the same experimenter.

### Eyeblink conditioning - habituation to eyeblink conditioning apparatus

Mice were head-fixed onto the head bar and allowed to walk on the treadmill for 20–30 min per day for 2 days without any stimuli, to get them acquainted with the eyeblink set-up.

### Eyeblink conditioning - baseline - finding the proper tone threshold for each animal

After 2 days of habituation, we measured the sensitivity of each mouse to the CS+, the CS−, and all other tone frequencies used to test generalization, which consisted in tones ranging from 2 to 8 kHz and 12-20 kHz in steps of 2 kHz spacing distance between each other. Within these non-reinforced tone frequencies, we also included some with closer spacing difference of 5 and 10% in both higher and lower directions from the CS+ (9, 9.5, 10.5 and 11 kHz). We choose to add these frequencies because previous studies have shown that some mice are able to discriminate between sounds as close as 2% apart (de Hoz and Nelken, 2014) and our interest is in the effect of the tonal spacing difference on both learning and generalization.

Since the responsiveness of an individual mouse to auditory stimuli can slightly vary from day to day, we repeated this measurement for 10 consecutive days for each animal (30 min each day). Each baseline session consisted of 2 blocks of 10 sounds only (i.e., tones that were never reinforced) and 2 airpuff only (US only) trials. Rodents’ auditory startle reflexes are behavioral indicators of their sensitivity to sounds (Boele, 2010). Eyelid responses were considered as startles following quantification of the velocity signal (1st derivative of the eyelid position signal) when peaks between 30 and 80 ms after the CS onset were larger than 3 standard deviations of the 500 ms baseline period and larger than an arbitrary threshold at 0.00025 (after traces have been normalized in a range of 0 as eye fully open and 1 as eye fully closed – see: *Eyeblink conditioning – data analysis*). At the end of the baseline sessions, we identified the highest sound pressure level (dB SPL) for all tone frequencies for each animal which would elicit limited amount of alpha startle responses.

### Eyeblink conditioning - acquisition differential training sessions

Mice were trained for 18 consecutive days (40 min/day). Each daily session was composed of 7 blocks of 31 trials each presented in a randomized fashion. Each block consisted of 1 US-only trial, 15 CS+ trials (reinforced), and 15 CS− trials (not-reinforced) (both CS− and CS+ trials had an interstimulus interval of 250 ms) (Figure 1B). Mice in the current experiment were overtrained due to the ratio of reinforced and non-reinforced trials (in our case 1:1). Mice were exposed to 105 CS− trials and 105 CS+ trials during each training session.

The CS+ was always a 10 kHz tone frequency with a duration of 280 ms co-terminating with an air-puff to the eye of 30 ms. The CS− trials were tone frequencies of 4 kHz for Grp.10CS + 4CS−, 9 kHz for Grp.10CS + 9CS− and 9.5 kHz for Grp.10CS + 9.5CS− with a duration of 250 ms.

### Eyeblink conditioning - generalization test sessions

The day after acquisition training ended, we tested stimulus generalization on days 19–22 (Figure 1B). Generalization test sessions lasted for 4 days, during which we presented all the tone frequencies (2 kHz, 4 kHz, 6 kHz, 8 kHz, 9 kHz, 9.5 kHz, 10 kHz, 10.5 kHz, 11 kHz, 12 kHz, 14 kHz, 16 kHz, 18 kHz, 20 kHz) of which the volume had been calibrated during the baseline to animals of all groups. Each session was composed of 8 blocks of 31 trials each presented in a randomized fashion. Each block of the generalization test session included 5 CS+ trials (reinforced), 1 US-only, and 5 tone-only trials of non-reinforced frequencies (including the respective CS− from each group) presented in random order.

### Eyeblink conditioning - data analysis

Individual eyeblink traces were analyzed with a custom-written MATLAB script (R2018a, Mathworks). First, the 2000 ms eyeblink traces were imported from our MySQL database into MATLAB. The trials were aligned at zero at the 500 ms pre-CS baselines. Trials with significant activity in the 500 ms pre-CS period (>7 times the interquartile range), were regarded as invalid for further analysis. We calculated the percentage of rejected trials in total which was around 10 to 15% for each mouse and each session. The signal was normalized so that the size of a full blink was 1 and the eyelid fully open corresponded to a value of 0 (fraction eyelid closure, FEC). The normalization to full blink 1 was done by dividing each trace for the averaged UR value that was calculated over *all* eyelid traces in both US only and CS+ trials for one session.

In our analysis, we included both CS+ and CS− trials, because we were interested in the difference between the profile of the conditioned response (CR) of the 10 kHz CS+ and the CR to the CS− for Grp.10CS + 4CS−, Grp.10CS + 9CS−, and Grp.10CS + 9.5CS− trials. We counted mice CRs during differential training across both CS+ and CS− trials, while only across CS− during the generalization test period. In valid normalized CS+ and CS− trials, an eyelid response was considered as a CR when the maximum amplitude between 150 and 249 ms after CS onset was larger than 0.05 and a positive slope was present in the 150 ms before the time point where the US would have been delivered (US is omitted in CS−only trials).

For the analysis, we considered all trials that did and did not show startle alpha responses and determined for each mouse for each session the percentage of trials in which a CR was present, which we will refer to as ‘CR percentage’. For each trial, we determined the maximum fraction eyelid closure (FEC) – as ‘eyelid closure – all trials’, between 150 and 249 ms after CS onset for each CS− and CS+ trials (therefore, excluding the eyelid peak due to the US delivery). In addition, in trials wherein a CR was present, we determined the maximum amplitude referred to as ‘eyelid closure – CR trials’, in the same above-mentioned time intervals. Finally, we investigated CR adaptive timing during the generalization test sessions with the latency to the onset of the CR relative to CS onset, and we refer to it as ‘latency to CR onset’, and the latency to maximum eyelid closure relative to CS onset, as ‘latency to CR peak’. Latency to CR onset was the only measure computed only for trials wherein no alpha startle response was present.

Statistical analysis was done using multilevel linear mixed-effects models (LME) in R Studio using the *nlme* package (code available upon request). In our main analysis, session, experimental group, and tone frequency (either CS+ or CS− for the differential training sessions or tone test frequencies for the generalization test sessions) were considered as fixed effects, and mouse was considered as a random effect. LMEs have several major advantages over standard parametric and non-parametric tests (Aarts et al., 2014; Schielzeth et al., 2020): (1) LMEs are robust to violations of normality assumptions, which is often the case in biological data samples; 2) LMEs can handle heteroscedasticity; (3) LMEs, like no other test, take into account the nested data structure, taking into account all data and their intra-class correlation; and (4) LMEs are better in handling missing datapoints than (RM-) ANOVAs. In our analyses, goodness of fit was determined by log likelihood ratio, BIC, and AIC scores. The distribution of residuals was inspected using Q-Q plots. Data was considered significant if the *p*-value was smaller than 0.05 after correcting for multiple comparisons using the FDR method.

## Results

We used Pavlovian eyeblink conditioning to test stimulus generalization following a differential training paradigm. First, mice were randomly divided into three groups. For each group during differential training, we used the same reinforced tone frequency always paired with an air-puff (CS+) and one different non-reinforced tone never paired with an air-puff (CS−). The non-reinforced CS was 4 kHz for mice in Grp.10CS + 4CS−, 9 kHz for mice in Grp.10CS + 9CS−, or 9.5 kHz for Grp.10CS + 9.5CS−. Before the differential training started, mice underwent auditory brainstem responses (ABRs) and a period of baseline to check their sensitivity in response to tones used during differential training and generalization test.

### Auditory brainstem and auditory startle responses

We tested both hearing threshold and startle response threshold of single subjects using auditory brainstem responses (ABRs) and auditory startle responses (ASRs). We performed ABRs before the start of differential training with Pavlovian eyeblink conditioning to ensure that animals did not have severely impaired hearing conditions. ABRs were recorded following tone pips presented at 4, 8, 16 and 32 kHz (see: standardized ABRs protocol Willott, 2006; Akil et al., 2016). Our B6CBAF1/J mice showed on average ABR responses at the lowest frequency of 4 kHz to tones with a sound pressure level (SPL) of around 38 dB. For 8 and 16 kHz, ABR peaks were elicited with the lowest sound pressure level of 22 dB SPL and 13 dB SPL, respectively. We found the highest thresholds of around 43 dB SPL on average in response to tone pips at 32 kHz (Supplementary Figure 1; Supplementary Table 1). We found no effect of group on ABR thresholds (*F* (23, 2) = 0.36, *p* = 0.69; Supplementary Figure 1; Supplementary Table 1). Our results are in line with previous measurements on the CBA/CaJ strain at 9 weeks of age done by Zheng et al. (1999) who reported ABR thresholds of 34, 23, 15 and 40 dB SPL for the 4 kHz, 8 kHz, 16 kHz and 32 kHz stimuli, respectively. Since we found comparable ABR thresholds between our B6CBAF1/J mice and the CBA/CaJ strain, we conclude that hearing was intact in our animals. The same ABRs were recorded at the end of the experiment (Data not shown, available upon request) to confirm hearing threshold of the animals. Subsequently, we tested for each mouse the sound levels at which an auditory startle response (ASR) occurred across the frequency range. Auditory startle responses in rodents are characterized by a rapid contraction of skeleton and facial muscles (Pantoni et al., 2020), which also determines a partial eyelid closure. Sometimes a late component of these eyelid startle responses called B-startle can mask or mimic a cerebellar CR (Boele, 2010, p. 202). This is the reason why it was fundamental to precisely identify the SPL and frequency for each mouse that would elicit minimal or no B-startle responses. For the quantification of the alpha startle responses we used the velocity signal (1st derivative of the eyelid position signal). The alpha startle response was present when peaks between 30 and 80 ms after the CS onset were larger than 3 standard deviations of the 500 ms baseline period and larger than an arbitrary threshold at 0.00025 (after traces have been normalized in a range of 0 as eye fully open and 1 as eye fully closed). All sound stimuli had the same duration and ramp/decay pattern and were never reinforced with an air-puff to the eye during baseline sessions. The baseline sessions were repeated for 10 days, each day consisting of 20 trials. During baseline, all the animals received the exact same number of trials consisting of only tones, so that we avoid the potentially differentiating effect of latent inhibition (Lubow and Moore, 1959; Lubow, 1973). Measurements on the last day of baseline showed considerable variation in startle threshold within each group of mice in response to tone only trials, although response thresholds within each mouse were uniform so that more sensitive mice tended to be more sensitive to all frequencies (Supplementary Figure 2; Supplementary Table 2). In general, our B6CBAF1/JRj mice showed similar sensitivity to all tested tone frequencies, as 61 dB was the lowest SPL detected when all dB SPLs were averaged across all animals, compared to the previously measured 60 dB for the C57Bl6/J mice from our laboratory (Fiocchi et al., 2022). Interestingly, animals from Grp.10CS + 4CS− and Grp.10CS + 9CS− were less sensitive than Grp.10CS + 9.5CS− (Supplementary Figure 2; Supplementary Table 2). At the end of the baseline, we established proper SPLs for each mouse and for each stimulus, which were eliciting minimal alpha startle responses. We used these SPLs for the whole duration of the eyeblink conditioning differential training (day 1–18) and generalization test (day 19–22).

### Eyeblink conditioning – differential training

After baseline, all animals were conditioned for 18 consecutive days (1 session/day) using a 10 kHz tone (CS+), which was always reinforced with an air-puff directed to the eye (US), as well as another random tone (CS−), which was *not* reinforced with the US. Animals with a learning rate less than 5% following quantification of CR percentage between day 1 and 18 of training were excluded from the analysis. Note that two animals from Grp.10CS + 9.5CS− were excluded from the analysis of the tone generalization test sessions (as they passed away later on during the experiments) and were not considered in the stimulus generalization plots (therefore, for Grp.10CS + 9.5CS− *n* = 6; for other groups, *n* = 8).

**Figure 2:**
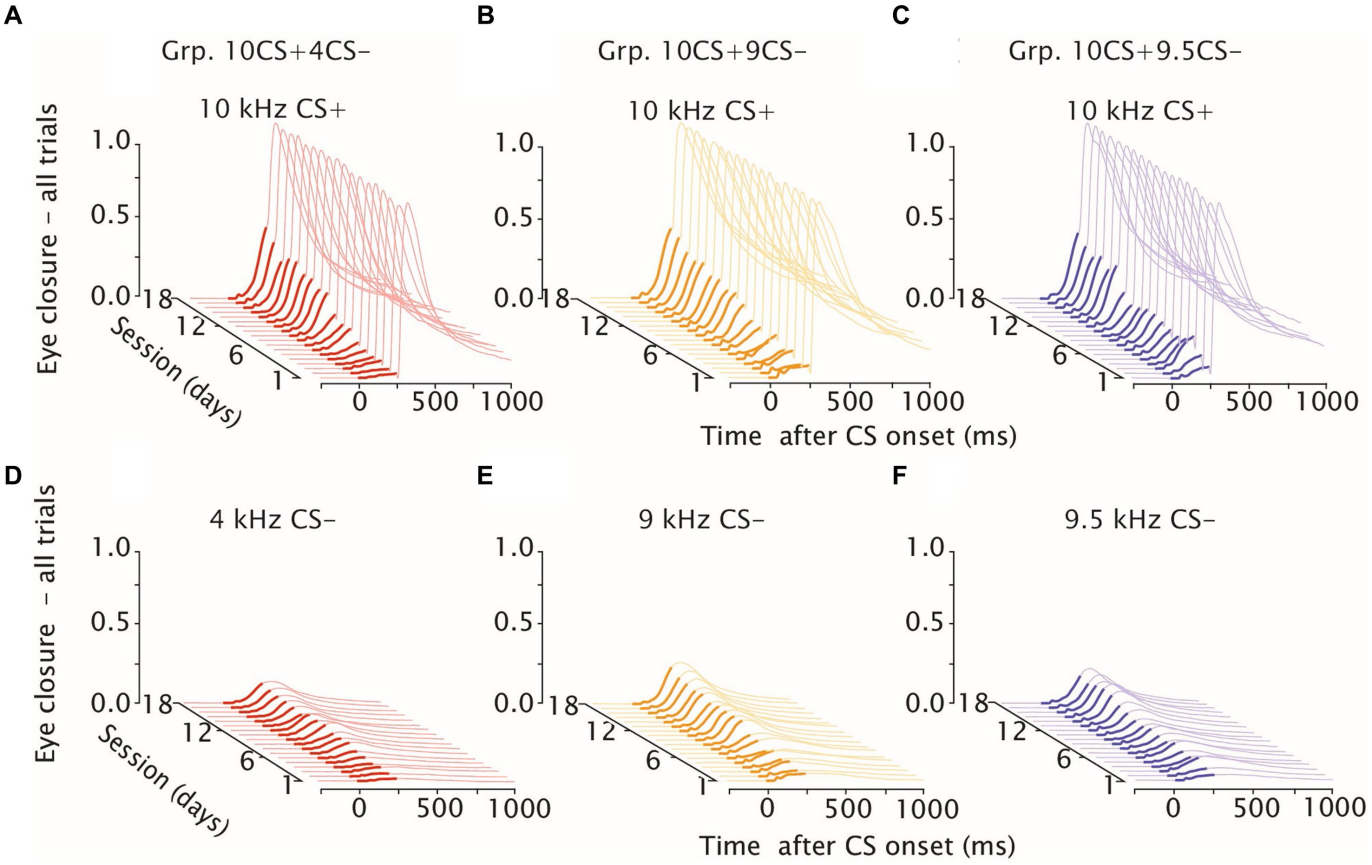
Eyelid closure during differential training. Waterfall plots of eyeblink traces during differential training, day 1–18. **(A)** Mice averaged eyelid response traces for Grp.10CS + 4CS− (in red) over 18 consecutive training sessions to the reinforced CS+. **(B,C)** same as **(A)** but showing eyelid responses, respectively, for Grp.10CS + 9CS− (in yellow) and Grp.10CS + 9.5CS− (in blue). **(D)** Mice averaged eyelid response traces for Grp.10CS+4CS− (in red) over 18 consecutive training sessions to the non reinforced CS-. **(E, F)** same as **(D)** but showing eyelid responses, respectively, for Grp.10CS+9CS− (in yellow) and Grp.10CS+9.5CS− (in blue). In each plot, thicker eyelid trace lines indicate CS onset and duration. Note that groups Grp.10CS + 4CS−, Grp.10CS + 9CS−, *n* = 8 and Grp.10CS + 9.5CS−, *n* = 6. All groups show consistent acquisition of eyelid responses to the 10 kHz CS+, and lower amplitude of eyelid closure at the end of training in response to the respective CS−.

We analyzed average traces of eyelid responses in both CS+ and CS− trials for each group over 18 days of training. For CS+ trials, we measured the eyelid response before the onset of the air-puff and noticed that this was progressively increasing over the course of sessions for all groups (Figures 2A–C). In a similar way, average eyelid responses to CS− trials increased over the course of training. However, CS− eyelid responses grew less and not as rapidly as the CS+ in all groups. This was evident, as amplitude of the fraction eyelid closure all trials (FEC) in response to the CS− at session 18 of differential training did not reach on average 0.20 for all groups, whereas it was around 0.42–0.45 in response to the CS+ (Supplementary Table 3; Figures 2D–F).

### CR percentage

**Figure 3:**
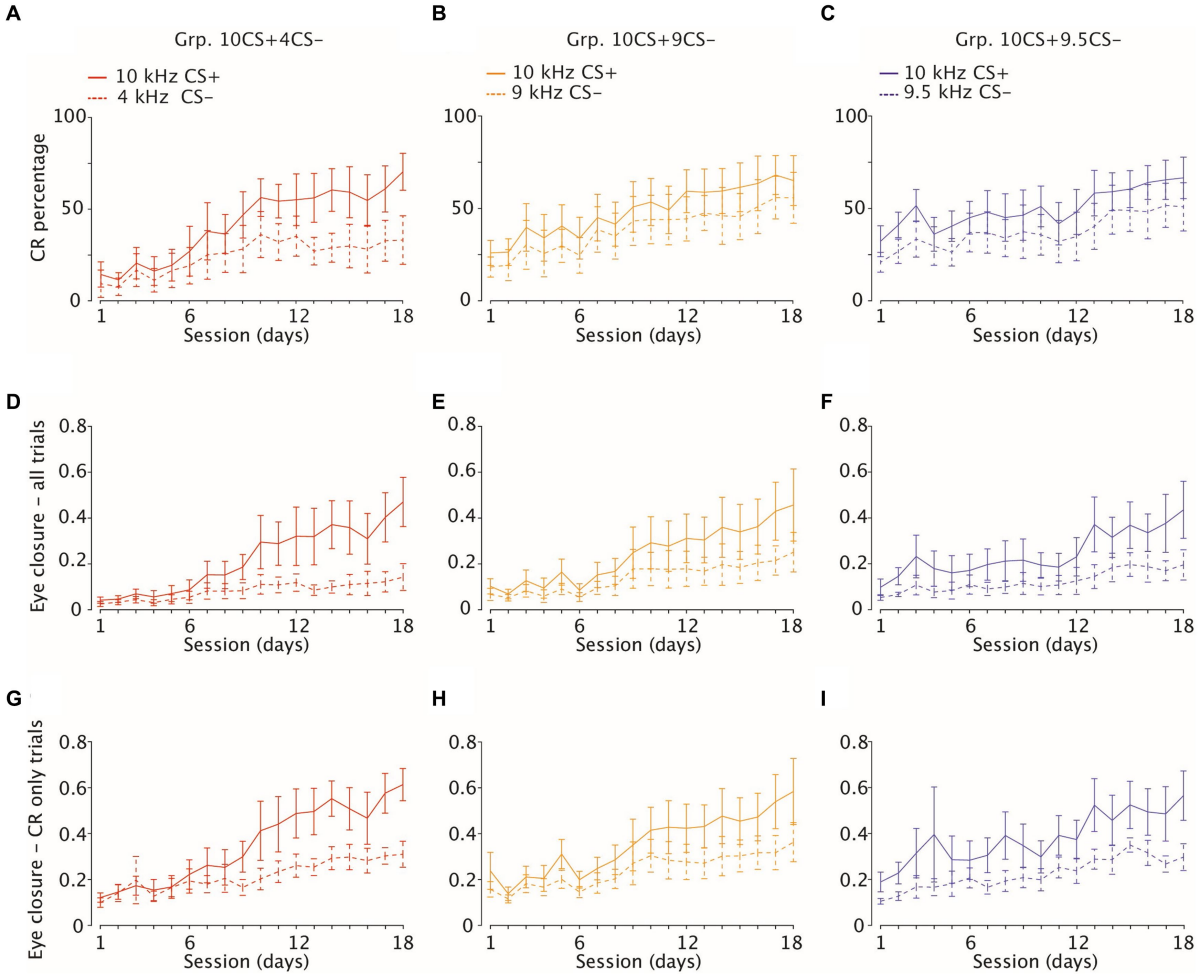
Acquisition of eyelid CRs and amplitude of eyelid closure in CS-only and CR-only trials. **(A–C)** Percentage of CRs (average and 95% CI) in response to reinforced (solid lines, CS+) and to non-reinforced (dotted lines, CS−) stimuli over 18 consecutive learning sessions (1 session/day) on the *x*-axis. Each panel is color-coded per group. Group 10CS + 4CS− *n* = 8 (in red), Group 10CS + 9CS− *n* = 8 (in yellow) and Group 10CS + 9.5CS− *n* = 6 (in blue) **(D–F)** Amplitude of eyelid closure over all trials **(G**–**I)** same as **(D–F)**, but considering eyelid closure over CR only trials.

We found a statistically significant effect of session on the average increase of CR percentage in response to the CS+ for all groups: Grp.10CS + 4CS−, Grp.10CS + 9CS− and Grp.10CS + 9.5CS− (Figures 3A–C; Supplementary Table 3; *F* (17, 119) = 6.65, *p* < 0.0001, *F* (17, 119) = 5.05, *p* < 0.0001 and *F* (17, 107) = 1.96, *p* = 0.019, respectively; ANOVA on LME). In a different way, the CR percentage to CS− significantly increased during differential training only for Grp.10CS + 4CS− and Grp.10CS + 9CS− (Figures 3A–C; Supplementary Table 3; *F* (17, 119) = 2.62, *p* = 0.0012 and *F* (17,119) = 3.93, *p* < 0.0001; ANOVA on LME). We did not find a significant effect of session for CS− of Grp.10CS + 9.5CS− (Figures 3A–C; Supplementary Table 3; *F* (17, 107) = 1.33, *p* = 0.186; ANOVA on LME).

The main purpose of our differential training was to establish how well mice could discriminate between sounds when one was positively reinforced (CS+) and another one was not (CS−). For this reason, we compared CR probability between CS+ and CS− for each group of mice and found that this was always significantly higher for CS+ (Figures 3A–C; Supplementary Table 3; for Grp.10CS + 4CS−, Grp.10CS + 9CS− and Grp.10CS + 9.5CS−: *F* (1, 245) = 55.49, *p* < 0.0001; *F* (1, 245) = 25.04, *p* < 0.0001; *F* (1, 221) = 34.91, *p* < 0.0001, respectively; ANOVA on LME). However, post-hoc analysis only revealed a significant difference between CS+ and CS− for Grp.10CS + 4CS− starting around session 13, while there was never an effect for Grp.10CS + 9CS− or Grp.10CS + 9.5CS− (Figures 3A–C; Supplementary Table 3). On the last day of training (day 18), animals from Grp.10CS + 4CS− reached the highest CR percentage in response to the reinforced CS+ of 70.29 (±10.0), while for Grp.10CS + 9CS− and Grp.10CS + 9.5CS− this was 65.64 (±13.5) and 67.08 (±11.3), respectively. The CS− percentages were 33.13 (±13.2) for Grp.10CS + 4CS−, 56.26 (±13.8) for Grp.10CS + 9CS−, and 51.25 (±13.1) for Grp.10CS + 9.5 CS−. These data indicate that there was a difference of around 30% in the amount of CRs on the last day of training between CS+ and CS− trials for animals of Grp.10CS + 4CS−, while this difference was slightly less pronounced (around 10–15%) for Grp.10CS + 9CS− and Grp.10CS + 9.5CS− (Supplementary Table 3; all values: mean ± 95% CI).

To determine whether there was a statistically significant difference between groups on CS+ response probabilities, we ran a linear mixed-model (LME) using group, session, and group*session as fixed effects and mouse as random effect. This analysis revealed a main effect of session on CR probability in response to the CS+ (Supplementary Table 3; *F* (17, 345) = 12.33, *p* < 0.0001; ANOVA on LME), but no significant effect of group (Supplementary Table 3; *F* (2, 21) = 0.713, *p* = 0.501; ANOVA on LME) nor an interaction effect of group*session (Supplementary Table 3; *F* (34, 345) = 1.01, *p* = 0.324; ANOVA on LME).

### Fraction eyelid closure amplitude

Further quantification of fraction eyelid closure (FEC) across *all trials* revealed a significant increase across sessions in response to CS+ for all groups (Figures 3D–F; Supplementary Table 3; for Grp.10CS + 4CS−, Grp.10CS + 9CS− and Grp.10CS + 9.5CS−: *F* (17, 13811) = 227.52, *p* < 0.0001; *F* (17, 13277) = 188.30, *p* < 0.0001; *F* (17, 12459) = 88.90, *p* < 0.0001, respectively; ANOVA on LME). The average amplitude of CS− trials also increased during differential training within each group of animals (Figures 3D–F; Supplementary Table 3; for Grp.10CS + 4CS−, Grp.10CS + 9CS− and Grp.10CS + 9.5CS−: *F* (17, 27569) = 227.59 with *p* < 0.0001; *F* (17, 13128) = 106.20 with *p* < 0.0001; and *F* (17, 12356) = 51.38, with *p* > 0.0001, respectively; ANOVA on LME). Fraction eyelid closure was always found significantly higher in CS+ trials compared to CS− in all groups (Figures 3D–F; Supplementary Table 3; for Grp.10CS + 4CS−, Grp.10CS + 9CS− and Grp.10CS + 9.5CS−: *F* (17, 27551) = 3011.13 with *p* < 0.0001, *F* (1, 26362) = 1292.91, with *p* < 0.0001, *F* (1, 24822) = 1883.90 with *p* < 0.0001, respectively; ANOVA on LME). Post-hoc analysis revealed that the average FEC of the CS+ compared to the CS− showed a pattern of progressively earlier significant difference the more the CS+ and CS− were similar. Indeed, the CS+ started to be significantly higher around session 7 for Grp.10CS + 4CS− and session 5 for Grp.10CS + 9CS−, while it already showed significance on the first day of training for Grp.10CS + 9.5CS−. Interestingly, we found that fraction eyelid closure (FEC) grew differently in response to CS+ and CS−. Indeed, the CS+ on the last day of training (session 18) in all our groups measured between 0.43 and 0.46, but the CS− for Grp.10CS + 4CS− was lower (0.14 (±0.05)) compared to the amplitudes of Grp.10CS + 9CS− (0.24 (±0.08)) and Grp.10CS + 9.5CS− (0.18 (±0.06)).

Similar to CR percentage, we compared CS+ amplitude of FEC responses by running a linear mixed-model (LME) using group, session, and group*session as fixed effects and mouse as random effect. This analysis showed that there was not a significant effect of group on the CS+ FEC amplitude (Supplementary Table 3; *F* (2, 21) = 0.050, *p* = 0.951; ANOVA on LME), while there was an effect of session (Supplementary Table 3; *F*(17, 39497) = 464.27, *p* < 0.0001; ANOVA on LME) and of the session*group interaction (Supplementary Table 3; *F* (34, 34497) = 21.06, *p* < 0.0001; ANOVA on LME). Similarly to the CR percentage of the CS+, FEC amplitude of animals from Grp.10CS + 9.5CS− were less pronounced at the end of training compared to the other two groups, although this difference was not significant.

### CR amplitude

The amplitude of fraction eyelid closure computed on all trials is not a measure of the actual amplitude of the CR to tone only trials. For this reason, we also computed average amplitude considering *only trials which show a CR,* which we called CR amplitude. We calculated CR amplitude in CS+ trials and found a significant effect of session for each group (Figures 3G–I; Supplementary Table 3; for Grp.10CS + 4CS−, Grp.10CS + 9CS− and Grp.10CS + 9.5CS−: *F* (17, 5766) = 73.54 with *p* < 0.0001, *F* (17, 6479) = 103.97 with *p* < 0.0001, *F* (17, 6148) = 77.56 with *p* < 0.0001, respectively; ANOVA on LME). This was true also for CS− trials (Figures 3G–I; Supplementary Table 3; *F* (17, 9044) = 87.97 with *p* < 0.0001, *F* (17, 5167) = 45.17 with *p* < 0.0001, *F* (17, 4553) = 42.69 with *p* < 0.0001, respectively; ANOVA on LME). We also noticed that within each group, the CS+ eyelid responses which we could consider CRs were on average about 0.25–0.30 higher compared to CS− on the last day of training (day 18) (Supplementary Table 3).

Our results showed that CR amplitudes in CS+ were consistently higher compared to CS− in all groups (Figures 3G–I; Supplementary Table 3; respectively for Grp.10CS + 4CS−, Grp.10CS + 9CS− and Grp.10CS + 9.5CS−: *F* (1, 9026) = 984.17 with *p* < 0.0001, *F* (1, 11653) = 1192.1423 with *p* < 0.0001, *F* (110708) = 1606.12 with *p* < 0.0001, respectively; ANOVA on LME). Similar to FEC amplitude, following post-hoc analysis we found that CR amplitude for Grp.10CS + 4CS− and Grp.10CS + 9CS− started to show significant differences between CS+ and CS− at session 6, while Grp.10CS + 9.5CS− already showed a significant difference around session 2 during differential training (Supplementary Table 3).

Next, we computed a linear-mixed effect model (LME) using session, group, and their interaction as fixed effects and mouse as random effect. We did not find a significant effect of the group factor on the CS+ amplitude across *CR only* trials (Figures 3G–I; Supplementary Table 3; *F* (1, 21) = 0.44 with *p* = 0.645; ANOVA on LME). However, similar to the FEC amplitude there was a significant effect of session (Figures 3G–I; Supplementary Table 3; *F* (17, 18393) = 230.78 with *p* < 0.0001; ANOVA on LME) and interaction of group*session (Figures 3G–I; Supplementary Table 3; *F* (34, 18393) = 12.26 and *p* < 0.0001; ANOVA on LME).

### Eyeblink conditioning - stimulus generalization test

The day after the last session of training (day 18), we tested the generalization of the CS+ for four consecutive days (1 session/day) on the three groups of mice (in Figure 1B, days 19–22). During generalization test sessions, mice were exposed to tone frequencies never paired with an air-puff (2, 4, 6, 8, 9, 9.5, 10.5, 11, 12, 14, 16, 18, 20 kHz), as well as to the 10 kHz tone only and to the 10 kHz CS+ paired with an air-puff US. All the stimuli presented during the generalization test sessions had the exact same duration of 280 ms and ramp/decay times of 25 ms as for the CS+ and the CS− during differential training. For all groups, n = 8. Note that two animals died during the experiment for GR10CS + 9.5CS− so that during generalization test sessions *n* = 6.

### CR percentage

**Figure 4:**
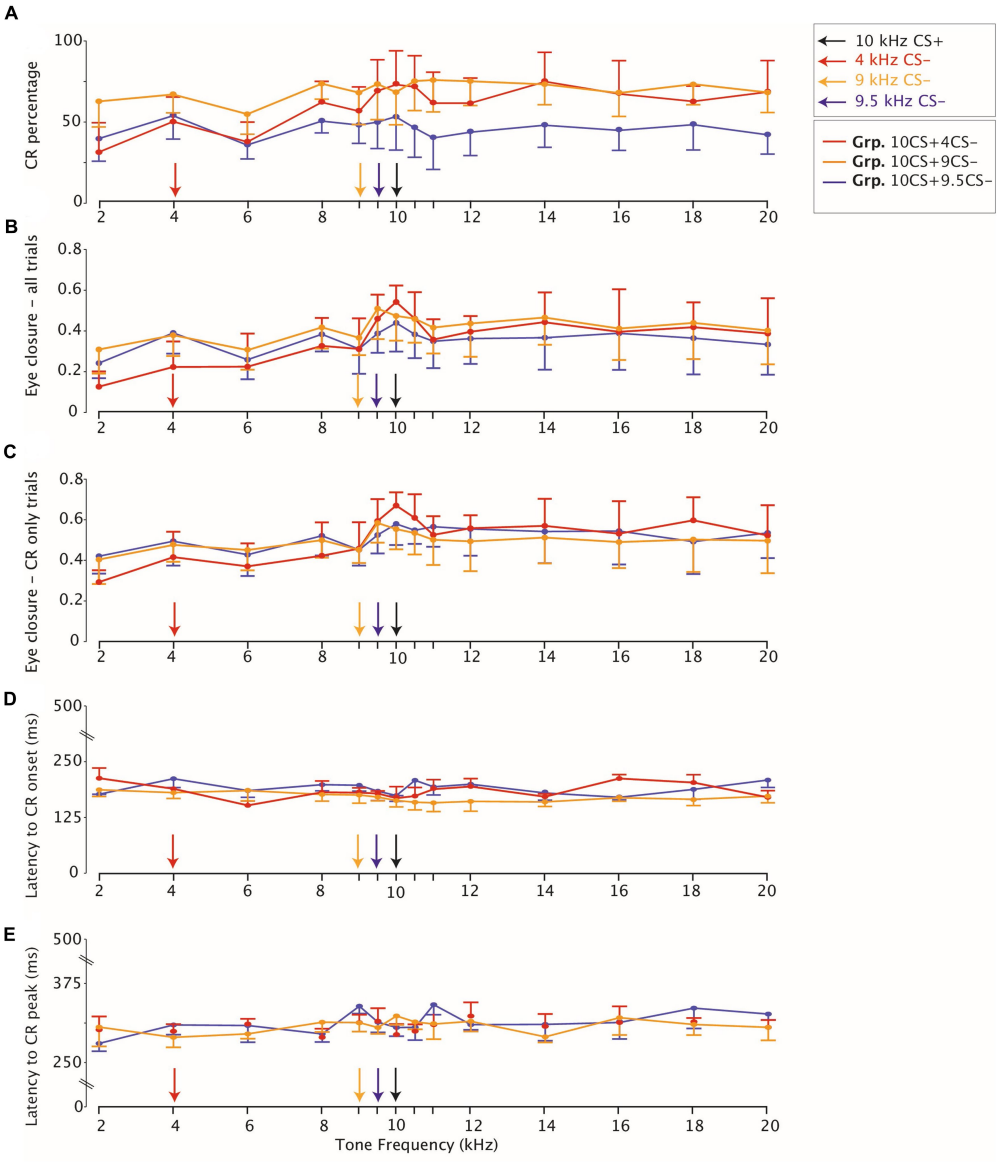
Eyelid closure testing stimulus generalization peaks for all groups around the trained frequency. **(A)** Averaged CR percentage in response to non-reinforced CS-only trials. The legend on the right on the top indicates that for each panel on the x-axis the CS- and the CS+ 10kHz are highlighted with arrows. The legend on the right below indicates the color coding for each group. CI at 95% indicates variation across animals for each group. **(B)** Eyelid closure over all trials **(C)** same as **(B)** but eyelid closure computed only over trials which show a CR. **(D)** Latency to CR onset and **(E)** Latency to CR peak are stable across tone generalization test frequencies.

We found a significant effect of tone generalization on the average of eyelid CRs for Grp.10CS + 4CS− (Figure 4A; Supplementary Table 4; *F* (13, 91) = 4.31, *p* < 0.0001; ANOVA on LME), but not for Grp.10CS + 9CS− and Grp.10CS + 9.5CS− (Figure 4A; Supplementary Table 4; *F* (13, 91) = 1,75, *p* = 0.063, and *F* (13, 65) = 1.13, *p* = 0.346 respectively; ANOVA on LME). More specifically, animals from Grp.10CS + 4CS− showed a downward gradient in the direction of lower frequencies than the CS+ (from 72.60 (±12.0)% for the 10 kHz to 30.39(±18.2)% for the 2 kHz), which was less evident for higher frequencies (71.22 (±8.8)% for 10.5 kHz and 67.66 (±13.8)% for 20 kHz) (Figure 4A; Supplementary Table 4; all values: mean ± 95% CI). On the other hand, animals from Grp.10CS + 9CS− and Grp.10CS + 9.5CS− showed a less steep decreasing gradient than Grp.10CS + 4CS−. Following post-hoc analysis, none of the comparisons between CS+ and tone frequencies tested during generalization showed significance within Grp.10CS + 9CS− and Grp.10CS + 9.5CS−. In addition, our results show that CR probability in Grp.10CS + 4CS− dropped off almost 40% from the CS+ to the 2 kHz, while Grp.10CS + 9CS− and Grp.10CS + 9.5CS− animals’ responses from the CS+ trials to the 2 kHz tone decreased around 10% in Grp.10CS + 9CS− and almost 15% in Grp.10CS + 9.5CS− (Supplementary Table 4).

Next, we ran a linear-mixed model (LME) using group, tone frequency, and group*tone frequency as fixed effect and mouse as random effect. We found that there was a statistically significant main effect of the tone frequency used on CR percentage (Supplementary Table 4; *F* (13, 247) = 5.22, *p* < 0.0001; ANOVA on LME), but no effect of group (Supplementary Table 4; *F* (2, 19) = 0.576, *p* = 0.571; ANOVA on LME), nor group*tone frequency interaction (Supplementary Table 4; *F* (26, 247) = 1.35, *p* = 0.123; ANOVA on LME).

### Fraction eyelid closure amplitude

**Figure 5:**
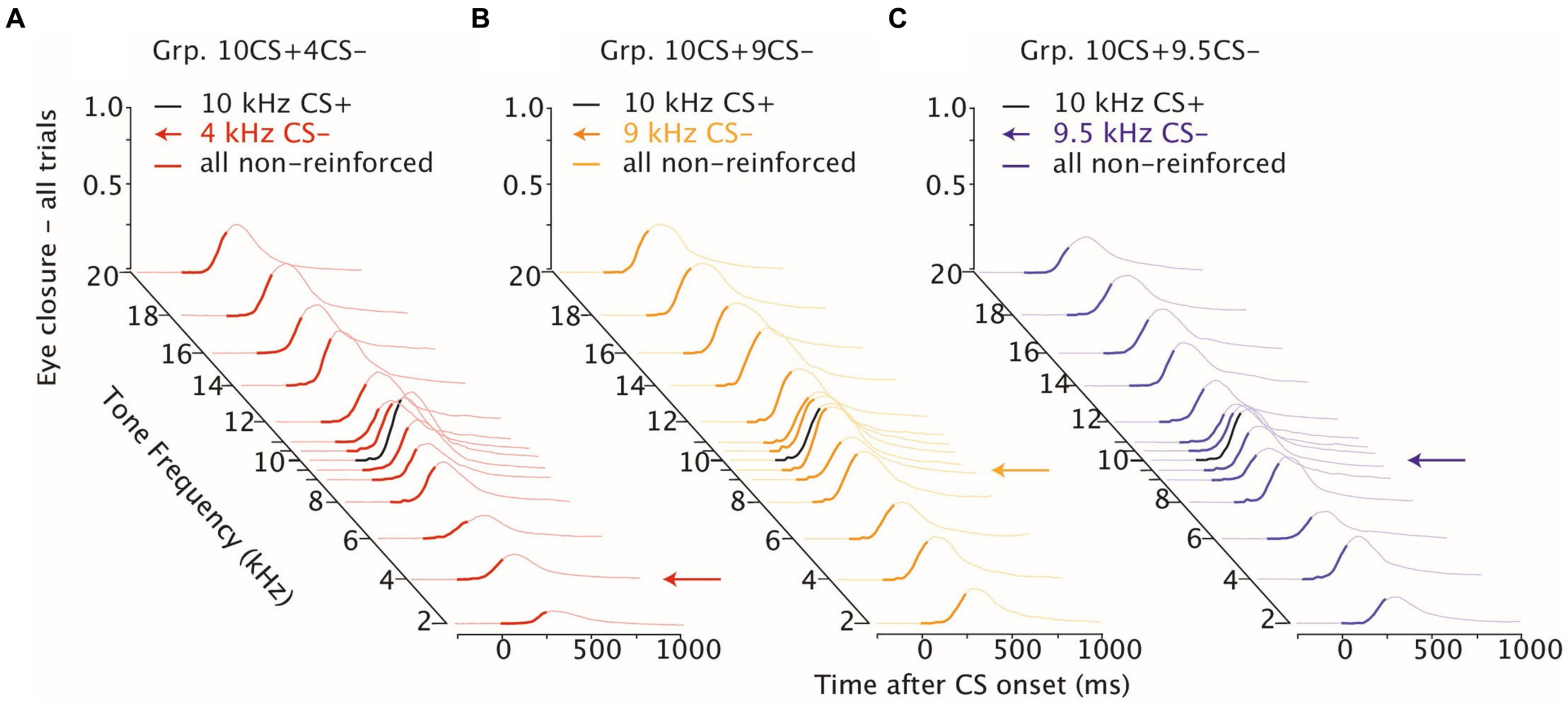
Stimulus generalization following differential training. Waterfall plot of eyelid responses to the non-reinforced generalization test frequencies color-coded per group. On the *x*-axis the time after CS onset is presented in milliseconds, on the *y*-axis the eye closure traces are averaged for each tone frequency used during stimulus generalization test sessions. For each plot the CS− is indicated with an arrow and the CS+ instead is colored in black. Note that GR10CS + 4CS− in red and GR10CS + 9CS− in yellow *n* = 8, while GR10CS + 9.5CS− in blue *n* = 6.

We found a similar effect of tone frequencies on the eye closure amplitude considering all trials in all groups (Supplementary Table 4; for Grp.10CS + 4CS−, Grp.10CS + 9CS− and Grp.10CS + 9.5CS−: *F* (13, 1841) = 21.36 with *p* < 0.0001, *F* (13, 1737) = 6.78 with *p* < 0.0001, *F* (13, 1375) = 3.64 with *p* < 0.0001, respectively; ANOVA on LME), as our data showed a decreasing gradient in the direction of lower frequencies than the CS+ (Figures 5A–C, 4B; Supplementary Table 4). Averaged FEC amplitude peaked at the CS+ with an amplitude of 0.53 (±0.16) for Grp.10CS + 4CS− (Figure 5A), 0.46 (±0.17) for Grp.10CS + 9CS− (Figure 5B), and 0.43 (±0.12) for Grp.10CS + 9.5CS− (Figure 5C). This amplitude decreased in response to a 2 kHz tone around 0.12 (±0.07) for Grp.10CS + 4CS−, 0.30 (±0.12) for Grp.10CS + 9CS−, and 0.23 (±0.08) for Grp.10CS + 9.5CS−. We found a similar decrease also in response to higher tone frequencies than the CS+ in each group of mice; for instance, the 20 kHz tone in Grp.10CS + 4CS− showed a FEC amplitude of 0.38 (±0.11), for Grp.10CS + 9CS− of 0.39 (±0.16), and for Grp.10CS9.5CS− of 0.32 (±0.07).

**Figure 6:**
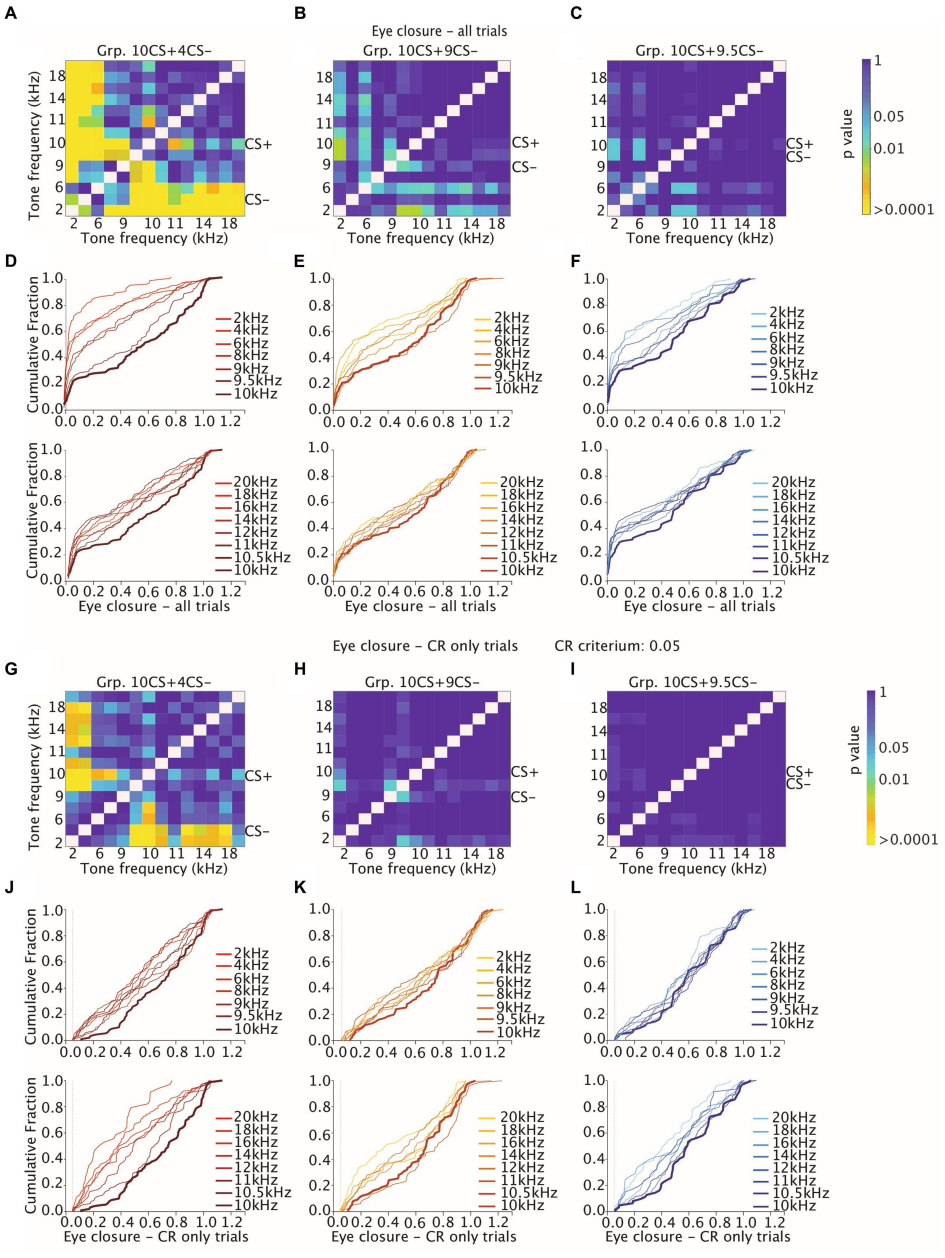
Heatmaps showing adjusted *p*-values of all tone-tone comparisons for cumulative distribution functions (CDFs) of amplitude of eyelid closure over all trials and over CR only trials. **(A–C)** Heatmaps measuring the effect of tone frequency on cumulative distribution of amplitude of eyelid closure over all trials for Grp10CS + 4CS− (left), Grp10CS + 9CS− (center), and Grp10CS + 9.5CS− (right). Color indicates the *p*-value. Note that the heatmap is on a logarithmic scale. All *p*-values were calculated using a Kolmogorov–Smirnov test on the cumulative distribution functions (CDFs). All *p*-values were adjusted for multiple comparisons using a False Discovery Rate (FDR). **(D–F)** Cumulative distribution function of eyelid closure calculated over all trials for different tone frequencies for each group. Different shades of color per group indicate distance between the CS+ and the other tone frequencies. Generalization stimuli were divided in two separate plots for tone frequencies higher (bottom) and lower (top) than the CS+. The CS+ is illustrated in both top and bottom plot as reference with a thicker line. **(G–I)** Same as **(A–C)**, but now considering the effect of tone frequency on cumulative distribution of amplitude of eyelid closure over CR only trials **(J–L)** same as **(D–F)**, but now considering the effect of tone frequency on amplitude of eyelid closure computed over CR only trials. Grp10CS + 4CS− (left), Grp10CS + 9CS− (center), and Grp10CS + 9.5CS− (right). Reference color bar on the right indicates the level of significance for the *p*-values that are in the figures (0.01, 0.05 and > 0.0001). CR criterium was established at 0.05. For complete statistics we refer to Supplementary Table 5.

These data indicate that for Grp.10CS + 4CS− there was a steeper downward gradient in the direction of both lower and higher tones than the CS+, compared to Grp.10CS + 9CS− and Grp.10CS + 9.5CS− (Supplementary Table 4; Figure 4B; all values: mean ± 95% CI). Post-hoc comparisons revealed that FEC amplitudes for tone frequencies at least 10% higher and lower than the CS+ were significantly different from the 10 kHz used to test stimulus generalization. Interestingly, there was a significant difference in the FEC amplitude between the CS+ and the respective CS− in Grp.10CS + 4CS− and Grp.10CS + 9CS− (Supplementary Table 4; *p* < 0.0001 and *p* = 0.003, respectively), but not for Grp.10CS + 9.5CS− (Supplementary Table 4; *p* = 0.83). Linear-mixed effect models (LMEs) using group, tone frequency, and group*tone frequency as fixed effects and mouse as random effect, showed that there was a significant effect of tone frequency (Supplementary Table 4; *F* (13, 4953) = 26.68, *p* < 0.0001; ANOVA on LME) and interaction between group*tone frequency (Supplementary Table 4; *F* (26, 4953) = 3.48, *p* < 0.0001; ANOVA on LME). However, there was not a statistically significant effect of the group factor (Supplementary Table 4; *F* (2, 19) = 0.21, *p* = 0.805; ANOVA on LME). Comparison of the cumulative distributions of FEC amplitudes for each group revealed significant effects only for many of the sound frequencies used to test generalization of Grp.10CS + 4CS− (Figures 6A,D; For *p* values we refer to Supplementary Table 5; all Kolmogorov–Smirnov tests with correction for multiple comparison using FDR). In the present work, we used *p* values comparisons to extract information regarding the probability of obtaining the observed result. Specifically, we verified the assumption that similarity of non reinforced conditioned stimuli (CS−) to the reinforced stimulus (CS+) during differential training for Pavlovian eyeblink conditioning had an effect on stimulus generalization. Given that *p*-values offer a standardized way to report and compare results across different groups and their responses during stimulus generalization, we used *p* values heatmaps to explore this hypothesis. Cumulative distributions from Grp10CS + 9CS− and Grp10CS + 9.5CS− instead (Figures 6B,C,E,F) show a decreasing pattern with some significant differences for lower tones than the CS+ (For *p* values we refer to Supplementary Table 5), but not for higher tones.

### CR amplitude

We found a significant effect of tone generalization on the amplitude of trials showing a CR (both CS+ and CS−) in all groups (Supplementary Table 4; Figure 4C; for Grp.10CS + 4CS−, Grp.10CS + 9CS− and Grp.10CS + 9.5CS−: *F* (13, 1066) = 9.28 with *p* < 0.0001, *F* (13, 1228) = 4.22 with *p* < 0.0001, *F* (13, 839) = 2.29 with *p* = 0.005, respectively; ANOVA on LME). More specifically, the average CR amplitude for Grp.10CS + 4CS− clearly peaked at the CS+ (0.66 (±0.11)) and showed a downward gradient in both directions of higher and lower frequency tones (Supplementary Table 4). On the other hand, Grp.10CS + 9CS− and Grp.10CS + 9.5CS− did not show a clear generalization gradient, although the CR only amplitude peaked at the CS+ for animals in both these groups (Supplementary Table 4, respectively 0.55 (±0.16) and 0.57 (±0.09), all values: mean ± 95% CI). We also analyzed these data using group, tone frequency, and group*tone frequency as fixed effects and mouse as random effect in a linear-mixed effect model (LME). We found a significant main effect of tone frequency (Supplementary Table 4; *F* (13, 3133) = 12.07, *p* < 0.0001; ANOVA on LME) and also of the interaction tone frequency*group (Supplementary Table 4; *F* (26, 3133) = 1.99, *p* = 0.001; ANOVA on LME), while there was no effect of group on the amplitude of CR only trials (Supplementary Table 4; *F* (2, 19) = 0.128, *p* = 0.880; ANOVA on LME). In addition, we looked at the cumulative distribution of CR amplitude and found a significant difference between the CS+ and many of the sound frequencies used to test stimulus generalization in Grp.10CS + 4CS− (for *p* values we refer to Supplementary Table 5; All Kolmogorov–Smirnov tests with correction for multiple comparisons using FDR; Figures 6G,J), while all the other tone frequencies in Grp.10CS + 9CS− and Grp.10CS + 9.5CS− did not result in significant differences (Figures 6H,I,K,L).

**Figure 7:**
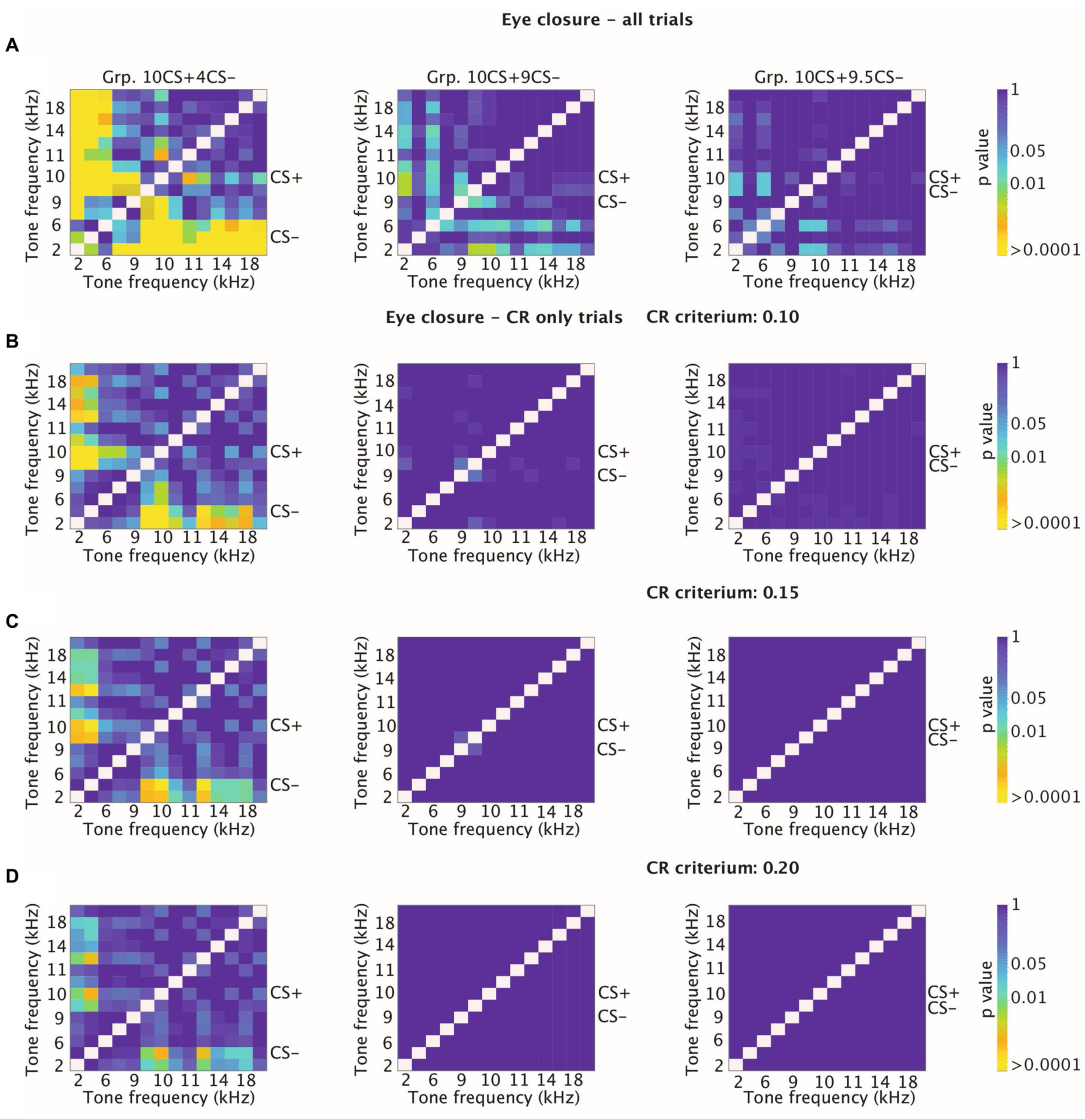
Heatmaps showing adjusted p-values for tone-tone comparisons for cumulative distribution functions (CDFs) of amplitude of eyelid closure over all trials and over CR only trials at different thresholds for CR detection. **(A)** Effect of sound frequency on cumulative amplitude of eyelid closure computed over all trials for Grp10CS + 4CS− (left), Grp10CS + 9CS− (center) and Grp10CS + 9.5CS− (right). For each group, CS+ and CS− are indicated on the side of the heatmap. Each heatmap illustrates on the *x*-axis and the *y*-axis tone frequencies used during the stimulus generalization test sessions. Color indicates the *p*-value from blue (less significant) to yellow (more significant). Reference color bar on the right indicates the level of significance for the *p*-values (0.01, 0.05 and > 0.0001). Note that the heatmap is on a logarithmic scale. All *p*-values were adjusted for multiple comparisons using false discovery rate (FDR). **(B)** Similar to panel **(A)**, but now computing cumulative amplitude of eyelid closure over CR-only trials using an arbitrary CR detection threshold at 0.10. **(C,D)** Similar to panel **(B)**, but now using a CR threshold of 0.15 and 0.20.

Previous work in mice showed that both CR probability and amplitude eyelid closure of CR only trials decreased on the degree of similarity between the tone frequency tested and the reinforced CS (CS+ in this experiment) (Fiocchi et al., 2022). However, this phenomenon was not found in rabbits (Khilkevich et al., 2018), which instead showed a relatively constant amplitude of eyelid closure CRs irrespectively of the tone frequency tested. For this reason, we also looked at higher CR thresholds other than the 0.05, which we used to detect CRs across training and generalization tests. More specifically, we plotted the cumulative distribution using higher CR thresholds of 0.10, 0.15 and 0.20 (Figures 7B–D). All in all, animals from Grp10CS + 4CS− showed significant comparisons between tone frequencies even when higher CR thresholds were tested (Figures 7A–D). Meanwhile, Grp10CS + 9CS− and Grp10CS + 9.5CS− did not show any significant comparison with any of the thresholds that were used (Figures 7A–D).

### CR peak time

One major advantage of the new technologies used to measure eyeblink conditioning is that these allow for measuring latency to the onset and the peak of the conditioned eyelid responses precisely. For this reason, we also analyzed measures of timing related to the onset and the peak latency of the eyelid CRs. We found a significant effect of tone frequency on the latency to CR peak for all our groups (Supplementary Table 4; for Grp.10CS + 4CS−, Grp.10CS + 9CS− and Grp.10CS + 9.5CS−: *F* (13, 1066) = 2.12 with *p* = 0.011, *F* (13, 1228) = 2.0 with *p* = 0.0177, and *F* (13, 839) = 3.49 with *p* < 0.0001, respectively; ANOVA on LME). On average, it appeared that lower and higher frequencies resulted in longer latencies to CR peak for animals in all groups (Supplementary Table 4; Figure 4E). When running post-hoc comparisons, we did not find any significantly different latencies to CR peak only when comparing CS+ and tones used to test stimulus generalization (Supplementary Table 4; Figure 4E).

All in all, our linear-mixed effect model (LME) revealed that there was a main effect of tone frequency on the latency to CR peak (Supplementary Table 4; *F* (13, 3133) = 3.35, *p* < 0.0001; ANOVA on LME) and of the interaction between tone frequency*group (Supplementary Table 4; *F* (26, 3133) = 1.96, *p* = 0.002; ANOVA on LME), while comparison between groups did not reveal a significant difference (Supplementary Table 4; *F* (2, 19) = 0.39, *p* = 0.681; ANOVA on LME).

### CR onset

In general, latency to CR onset was between 150 and 180 ms after the onset of the tone CS in all groups and there was an effect of generalization test tones (Supplementary Table 4; Figure 4D; for Grp.10CS + 4CS−, Grp.10CS + 9CS− and Grp.10CS + 9.5CS−: *F* (13, 209) = 2.23 with *p* = 0.009, *F*(13, 261) = 1.99 with *p* = 0.022, and *F* (13, 243) = 2.33 with *p* = 0.006, respectively; ANOVA on LME). Importantly, CR onset was computed across CR only trials that did not show a startle response (see: Methods, Eyeblink conditioning data analysis).

Next, from our linear-mixed effect model (LME) we analyzed the effect of tone frequency, group, and the interaction between tone frequency*group as fixed effects and mouse as random effect. Results from our LME showed that there was no significant effect of group (Supplementary Table 4; *F* (2, 19) = 1.65, *p* = 0.217; ANOVA on LME), but there was a significant effect for the interaction between tone frequency*group (Supplementary Table 4; *F* (26, 713) = 2.15, *p* = 000.8) and for the tone frequency (Supplementary Table 4; *F* (13, 713) = 2.28, *p* = 0.005; ANOVA on LME).

## Discussion

The main goal of our experiment was to investigate how discrimination affects generalization in the context of cerebellar learning using a well-established classical conditioning paradigm, Pavlovian eyeblink conditioning. Our findings show that the level of generalization in mice is affected following a differential training paradigm, when a non-reinforced (CS−) stimulus is presented during training of eyeblink CRs in response to one stimulus that is positively reinforced (CS+). According to our results, both CR probability and amplitude of eyelid closure across all trials decrease in response to lower tones than the CS+ irrespectively of the distance in frequency between the CS+ and the CS−, which are all at a lower frequency than the CS+ in this study. In addition, a wider tonal difference between the CS+ and the CS− (from 0.15 octave to 1.32 octaves tonal difference) made the generalization curve steeper in the direction of lower sounds than the CS+. We did find an effect on the latency of CR onset (Supplementary Table 4; *F* (13, 713) = 2.28, *p* = 0.005; ANOVA on LME) and also we observed a significant trend (Supplementary Table 4; *F* (13, 3133) = 3.35, *p* < 0.0001; ANOVA on LME) for a temporal shift of the CR peak.

### CR percentage

During differential training, we found a significant increase in CR percentage both in response to the CS+ and CS− for all groups of animals. This increase in CR percentage has also been found in both humans and rabbits during eyeblink differential training; the likelihood of conditioned responses to both CS+ and CS− in these species increased during initial phases, and responding to the CS+ continued to increase while responding to the CS− either stabilized or declined to a lower level (Gynther, 1957; Liu, 1971; Moore, 1972; Moore and Mis, 1973). Our animals of Grp10CS + 9.5CS− already showed a high CR percentage on day 1, which could be explained by the fact that the CS+ and CS− are very similar to each other in that the tone frequency that is reinforced and the one that is non-reinforced differ by only 5%. At the end of training, CR probability to the CS+ for animals from Grp10CS + 4CS− was higher compared to the other two groups, Grp10CS + 9CS− and Grp10CS + 9.5CS−. On the other hand, probability of CS− increased across sessions for both Grp10CS + 9CS− and Grp10CS + 9.5CS−, reaching over 50% at the end of training, while it remained around 30% for Grp10CS + 4CS−. We conclude that the CS+ and CS− similarity is inversely proportional to the increase in CS+ CR probability across sessions of differential training, while it is directly proportional to the increase in CS− probability. These data show that for mice learning a discrimination task between two tone stimuli is directly related to the similarity between CS+ and CS−. The proximity of the CS− to the CS+ did not directly affect our interpretation in this regard, as we investigated the hypothesis that different levels of discrimination affect generalization. Previous work from de Hoz and Nelken (2014) investigated principles of discrimination and generalization and established that mice could discriminate between frequencies that were as close as 7% supporting the frequencies that were chosen to establish differential training (i.e., 9 kHz CS− for 10% distance and 9.5 kHz CS− for 5% distance). We could speculate that using a CS− of either 7 kHz or 8 kHz would make the stimulus generalization gradient slightly steeper than the one observed for the 9 kHz and the 9.5 kHz for lower frequencies than the CS+. In addition, other factors could influence the differential training. For instance extending the training period, strengthening the intensity of the US or using higher frequency tones than the CS+ as non-reinforced stimuli could have an effect on the CR percentage and the level of discrimination. Indeed, it is possible that discrimination learning during eyeblink conditioning is weaker when the CS+ and CS− are close to each other because of the different frequency tuning of Purkinje cells in the cerebellar cortex and/or that of neurons in the cerebellar nuclei downstream. Recent findings highlight the heterogeneous nature of PCs responses and their consequent functional representation during simple associative behavior (De Zeeuw et al., 2023). Moreover, it should be noted that the complex spike activity of PCs during eyeblink conditioning can elicit a myriad of heterogeneous effects, both in the molecular layer and downstream (Badura et al., 2013; ten Brinke et al., 2015, 2017).

During the test of stimulus generalization, CR probability shows a clear decreasing gradient only when the spacing distance between the CS+ and CS− was at least 60% and the tone frequencies used were lower than the CS+. Our results show that tone-only trials with frequencies higher than the CS+ did not significantly reduce the percentage of eyelid responses. Previous studies on rabbits and eyeblink conditioning showed that CR probability peaked at the CS+ and decreased gradually after differential training when other stimuli are tested (Moore, 1964, 1972; Liu, 1971; Moore and Mis, 1973). However, none of these previous studies tested tone frequencies both higher and lower than the CS+. In relation to this matter, it could be argued that in order to complete the study of how discrimination affects generalization, future experiments should be addressed toward investigating how higher CS− than the CS+ affects stimulus generalization. In addition to that, it would be interesting to investigate PCs activity in response to CS+ and CS− during differential training. Our findings are partially in line with previous work done on stimulus generalization and eyeblink conditioning in rabbits (Moore, 1964, 1972; Liu, 1971; Moore and Mis, 1973), since we found a decreasing gradient of CR probability toward lower tone frequencies, which was particularly evident as the tonal difference between the CS+ and CS− was around 60%.

### Fraction eyelid closure amplitude/CR amplitude

During the test of stimulus generalization, similar to CR percentage, both the FEC amplitude and CR amplitude showed a stepwise decrease for lower tone-only trials than the CS+ in groups with either a 60% or 5% tonal difference between CS+ and CS−, as shown for Grp.10CS + 4CS− and Grp.10CS + 9.5CS−. On the other hand, when the difference between the CS+ and CS− was about 10%, there was not a clear gradient in FEC or CR amplitude response to lower tone-only trials. Thus in general, we did not find a clear pattern in response to higher tone-only trials. Previous studies in rabbits reported that FEC amplitude in stimulus generalization following non-differential training decreased with progressively different frequencies than the CS+ (Garcia et al., 2003; Ohyama, 2003). The same decreasing pattern was also found in mice following non-differential training (Fiocchi et al., 2022).

Looking only at the amplitude of the trials that showed a CR, we did not find a generalization gradient for Grp.10CS + 9CS− and Grp.10CS + 9.5CS−, while some sort of generalization gradient for test frequencies similar to the CS+ was evident in Grp.10CS + 4CS−. Previous experiments have shown that there is a decreasing pattern for the CR amplitude in mice and rabbits (Garcia et al., 2003; Fiocchi et al., 2022). However, Khilkevich et al. (2018) found that the amplitude of CR remained constant across tone-only trials used to test stimulus generalization in rabbits. These discrepancies can potentially be explained by considering not only the difference in the eyelid motor plant between mice and rabbits, but also by the performance level of the animals at the end of the training, which were overtrained in the Khilkevich et al. (2018).

### CR timing

Measures of adaptive timing in generalization of eyelid CRs following either differential or non-differential training have been ignored by most studies (Moore, 1964, 1972; Liu, 1971; Moore and Mis, 1973). Our experiments testing the lower frequency range of auditory tones show that CRs of the Grp10CS + 4CS− and Grp10CS + 9.5CS− mice appear to peak later on average compared to the CS+, while the CRs of the Grp10CS + 9CS− mice may peak earlier. That is, in our experiment a 10% difference between the CS+ and the CS− anticipates the latency of the CR compared to 60 and 5%. Previous studies on stimulus generalization have shown that after non-differential training mice CRs in response to tones used to test stimulus generalization peak later in response to higher frequencies than the one reinforced during training (Garcia et al., 2003; Fiocchi et al., 2022).

We found a main significant effect for CR onset of tone frequencies within each group after differential training, whereas there was no significant difference between the CS+ and the test tones used during stimulus generalization. Previous research shows that the CR onset of mice does not change when stimulus generalization is tested following non-differential training (Fiocchi et al., 2022) or when the duration of the CS is changed (Chettih et al., 2011). Garcia et al. (2003) instead reported an increased onset latency in rabbits, albeit it did not reach significance.

### Neural mechanisms

The circuitry underlying learning and memory formation of eyeblink CRs originates in the cerebellum at both the level of the Purkinje cells and cerebellar nuclei neurons (Mccormick et al., 1981, 1982a,b; Mauk and Donegan, 1997; Yeo and Hesslow, 1998; Mauk and Buonomano, 2004; Freeman and Steinmetz, 2011; Heiney et al., 2014; Freeman, 2015; ten Brinke et al., 2015, 2017). Both cell types are well designed to do so, as they receive signals about both the CS (via mossy fibers-parallel fibers pathway) and US (via climbing fibers) (Steinmetz et al., 1986, p. 198;Thompson and Steinmetz, 2009; De Zeeuw and Ten Brinke, 2015; De Zeeuw et al., 2021; Broersen et al., 2023).

One important characteristic of Purkinje cells is that they are constantly actively inhibiting the cerebellar nuclei (Jirenhed et al., 2017; Grasselli et al., 2020). During training of eyeblink CRs, the repeated pairing of the CS and US information at the Purkinje cell level results in a pause of the simple spike firing in response to the CS alone (Ohmae and Medina, 2015; ten Brinke et al., 2015; Jirenhed et al., 2017; Narain et al., 2018). As a consequence, the disinhibition of the cerebellar nuclei activity drives the emergence of eyeblink CRs (Foy et al., 1984; Berthier and Moore, 1986, 1990; Foy and Thompson, 1986; Krupa et al., 1990; Tracy, 1995; ten Brinke et al., 2017). Previous research has shown that cerebellar nuclei neurons also receive collaterals from mossy fibers, which respond specifically to the CS information and which are sufficient to drive the CR after learning (Boele, 2010; Boele et al., 2016; Broersen et al., 2023).

Neurons in the cerebellar nuclei have been found to respond selectively to a CS consisting of either a tone, a light, or a compound tone-light stimulus (Tracy et al., 2001; Broersen et al., 2023). Conversely, neuronal activity in the Purkinje cells evoked by auditory stimulation has so far not been found to show sensitivity to sound frequency (Radionova and Shmigidina, 1974; Altman et al., 1976). Results from our experiments show that both likelihood and amplitude of the Purkinje cell-driven CR change across groups when stimulus generalization is tested. Therefore, we hypothesize that the spacing distance between and CS+ generates a frequency-specific representation at the cerebellar nuclei level of the tones used during differential training, which may affect stimulus generalization even if CS+ and CS− are not prominently represented at the Purkinje cell level.

During differential training, the more the CS+ and CS− are similar to each other, the more the learning of the CRs might be reflected in the integrated responses of the cerebellar nuclei to the two different sounds. In addition, other brainstem regions may also contribute to sound processing and stimulus generalization, undergoing plasticity during this classical conditioning task at the same time.

The processing of auditory information engages several brainstem regions including the pontine nuclei, the main source of mossy fibers innervating the cerebellar cortex. The pontine nuclei, similar to other brainstem regions involved in CS information processing, show stimulus specificity and provide the mossy fiber input to the cerebellum with auditory, somatosensory and visual CS information (Steinmetz et al., 1986; Lewis et al., 1987; Steinmetz et al., 1987; Knowlton and Thompson, 1988; Steinmetz and Sengelaub, 1992; Tracy and Steinmetz, 1998; Hesslow et al., 1999; Bao et al., 2000; Freeman and Rabinak, 2004; Freeman et al., 2005). Leergaard and Bjaalie (2007) showed that pontine nuclei neurons largely maintain functional segregation in their projections. We could hypothesize that, within the auditory modality the mossy fibers carrying auditory information of the CS+ and CS− during differential training maintain frequency specificity from the pontine nuclei reaching the cerebellar nuclei through its collaterals (Broersen et al., 2023).

### Stimulus generalization and stimulus discrimination in fear learning and the role of cerebellum

The concept of stimulus generalization is important for our ability to constrain responses during our daily life. Mechanisms of generalization have been widely studied using fear conditioning as a form of classical conditioning. Especially in the context of fear learning, defensive responses are elicited by stimuli that predict an aversive event as a crucial hallmark of primate evolution (Dunsmoor and Paz, 2015). However, harmless stimuli can also be misinterpreted as dangerous and evoke defensive responses in contexts without any specific fear-related event. This mechanism of overgeneralization to non-threatening or irrelevant signals is a maladaptive mechanism and is considered a common denominal factor to anxiety disorders and posttraumatic stress disorder (PTSD) (Dunsmoor and Paz, 2015; Laufer et al., 2016).

Classical conditioning techniques have been used to investigate clinical fear and anxiety disorders in humans (Dunsmoor and Paz, 2015). Converging evidence is increasingly bringing attention to the role of cerebellum in both motor and non-motor domains, including fear learning (Maschke et al., 2003; Lange et al., 2015). Recent findings used the stimulus generalization test approach, so that after differential training between CS+ and CS−, stimulus generalization is tested on non-reinforced stimuli (including the CS+ and CS−). Lissek et al. (2008) developed a task using a perceptual dimension of increasing ring size to characterize generalization gradients of different anxiety disorders. Results from their experiments demonstrated that healthy subjects showed a steeper gradient of generalization, while anxiety patients strongly responded to non-reinforced stimuli, which were clearly dissimilar from the CS+ (Lissek et al., 2008). Understanding the role of differential training on the level of generalization represents a first step toward major understating of learning and memory formation mechanisms that are necessary for our survival.

## Data availability statement

The raw data supporting the conclusions of this article will be made available by the authors, without undue reservation.

## Ethics statement

The animal study was approved by EDC – Erasmus Laboratory Animal Science Center. The study was conducted in accordance with the local legislation and institutional requirements.

## Author contributions

FF: Writing – original draft, Visualization, Validation, Software, Resources, Project administration, Methodology, Investigation, Formal analysis, Data curation, Conceptualization. ND: Writing – review & editing, Software, Resources, Methodology, Investigation, Conceptualization. SD: Writing – review & editing, Writing – original draft, Supervision, Resources, Project administration, Methodology, Data curation, Conceptualization. MB: Writing – review & editing, Validation, Resources, Investigation, Formal analysis. AW: Writing – review & editing, Conceptualization. CZ: Writing – review & editing, Resources, Project administration, Methodology, Conceptualization. H-JB: Writing – review & editing, Resources, Project administration, Conceptualization.
