# Supplementary material for: Discrimination training affects stimulus generalization in mice during Pavlovian eyeblink conditioning
Source: Front Behav Neurosci. 2024 Aug 23;18:1446991. doi: 10.3389/fnbeh.2024.1446991 (PMC11377223; doi:10.3389/fnbeh.2024.1446991)
Supplement: Supplementary file 1 [file Data_Sheet_1.docx]

***Supplementary Material***

**Figure S1. Auditory Brainstem Responses (ABRs).** Single mice sensitivity to clicks of sound at 4, 8, 16 and 32 kHz were tested during ABRs showing normal auditory thresholds.

**Figure S2. Baseline single mice dB SPL per tone frequency (kHz).** Single mice dB per tone frequency (kHz) on the last day of baseline (Session 10). Animals from different groups show consistency in dB levels across tone frequencies (Supplementary Table 2). Boxes enclose the interquartile range (IQR) and whiskers show the minimum and maximum values excluding the IQR. Black thick line represents averaged dB values of all mice. Single mice dB are plotted on top of the boxplots for each tone frequency (kHz). Note that the scaling on the x-axis is not linear.

| **Mouse id.** | **4 kHz** | **8 kHz** | **16 kHz** | **32 kHz** | **Group** |
| --- | --- | --- | --- | --- | --- |
| **205** | 35 | 20 | 10 | 40 | **GR10CS+4CS-** |
| **207** | 30 | 15 | 0 | 30 |  |
| **209** | 30 | 10 | 5 | 40 |  |
| **258** | 40 | 30 | 25 | 60 |  |
| **260** | 50 | 25 | 20 | 50 |  |
| **262** | 50 | 30 | 20 | 50 |  |
| **267** | 40 | 25 | 25 | 60 |  |
| **637** | 40 | 30 | 15 | 35 |  |
| ***Mean*** | *39.37* | *23.12* | *15* | *45.62* |  |
|  |  |  |  |  |  |
| **204** | 35 | 15 | 10 | 30 | **GR10CS+9CS-** |
| **206** | 30 | 20 | 5 | 50 |  |
| **211** | 35 | 10 | 5 | 30 |  |
| **257** | 40 | 25 | 25 | 50 |  |
| **259** | 40 | 25 | 20 | 50 |  |
| **264** | 40 | 25 | 20 | 60 |  |
| **266** | 30 | 20 | 10 | 35 |  |
| **638** | 50 | 25 | 10 | 50 |  |
| **642** | 50 | 30 | 10 | 50 |  |
| ***Mean*** | *38.88* | *21.66* | *12.77* | *45* |  |
|  |  |  |  |  |  |
| **203** | 35 | 20 | 10 | 40 | **GR10CS+9.5CS-** |
| **210** | 25 | 10 | 5 | 30 |  |
| **256** | 35 | 20 | 10 | 40 |  |
| **261** | 35 | 20 | 10 | 50 |  |
| **263** | 40 | 25 | 25 | 50 |  |
| **265** | 40 | 30 | 25 | 50 |  |
| **639** | 50 | 20 | 5 | 35 |  |
| **640** | 40 | 25 | 10 | 35 |  |
| **641** | 35 | 25 | 10 | 40 |  |
| ***Mean*** | *37.22* | *21.66* | *12.22* | *41.11* |  |
|  |  |  |  |  |  |
| ***Mean Tot.*** | 38.46 | 22.11 | 13.26 | 43.84 |  |
|  |  |  |  |  |  |
| ***Group*** | *F(23,2)=0.36,p=0.69* | | | |  |
| ***Tone Frequency*** | *F(68,3)=196.91,p<.0001* | | | |  |
| ***Group*Tone Frequency*** | *F(69,6)=0.29,p=0.93* | | | |  |

**Supplementary Table 1| Auditory brainstem responses (ABRs) single subjects at tone frequencies of 4, 6, 8 and 32kHz.**

| **Mouse id.** | **2** | **4** | **6** | **8** | **9** | **9.5** | **10** | **10.5** | **11** | **12** | **14** | **16** | **18** | **20** |  |
| --- | --- | --- | --- | --- | --- | --- | --- | --- | --- | --- | --- | --- | --- | --- | --- |
| **205** | 39 | **43** | 15 | 48 | 33 | 30 | **38** | 43 | 29 | 25 | 43 | 43 | 50 | 50 | **GR10CS+4CS-** |
| **207** | 75 | **75** | 55 | 55 | 60 | 60 | **60** | 60 | 50 | 50 | 60 | 60 | 70 | 70 |  |
| **209** | 65 | **49** | 40 | 43 | 38 | 40 | **43** | 53 | 40 | 43 | 40 | 40 | 47 | 47 |  |
| **258** | 81 | **80** | 64 | 70 | 67 | 67 | **68** | 66 | 63 | 65 | 58 | 66 | 66 | 73 |  |
| **260** | 82 | **95** | 78 | 95 | 73 | 68 | **70** | 84 | 67 | 67 | 82 | 69 | 69 | 80 |  |
| **262** | 95 | **95** | 95 | 95 | 95 | 95 | **95** | 95 | 95 | 95 | 95 | 95 | 95 | 95 |  |
| **267** | 95 | **95** | 89 | 73 | 72 | 72 | **73** | 85 | 75 | 82 | 79 | 76 | 78 | 84 |  |
| **637** | 95 | **95** | 95 | 95 | 85 | 95 | **85** | 95 | 85 | 85 | 95 | 93 | 95 | 95 |  |
| ***Mean*** | 78.37 | **78.37** | 66.37 | 71.75 | 65.37 | 65.87 | **66.5** | 72.62 | 63 | 64 | 69 | 67.7 | 71.25 | 74.25 |  |
| Q1 | 72.5 | **68.5** | 51.25 | 53.25 | 54.5 | 55 | **55.75** | 58.25 | 47.5 | 48.25 | 54.25 | 55.75 | 62 | 65 |  |
| Q3 | 95 | **95** | 90.5 | 95 | 76 | 77.75 | **76** | 87.5 | 77.5 | 82.75 | 85.25 | 80.25 | 82.25 | 86.75 |  |
| **IQR** | 22.5 | **26.5** | 39.25 | 41.75 | 21.5 | 22.75 | **20.25** | 29.25 | 30 | 34.5 | 31 | 24.5 | 20.25 | 21.75 |  |
|  |  |  |  |  |  |  |  |  |  |  |  |  |  |  |  |
| **204** | 80 | 80 | 57 | 74 | **57** | 75 | **64** | 75 | 72 | 60 | 75 | 65 | 70 | 70 | **GR10CS+9CS-** |
| **206** | 55 | 53 | 33 | 47 | **35** | 30 | **37** | 47 | 40 | 37 | 43 | 43 | 60 | 60 |  |
| **211** | 45 | 43 | 25 | 55 | **37** | 38 | **38** | 53 | 40 | 33 | 47 | 32 | 57 | 57 |  |
| **257** | 70 | 63 | 50 | 40 | **51** | 44 | **55** | 49 | 52 | 49 | 50 | 42 | 57 | 52 |  |
| **259** | 95 | 95 | 95 | 95 | **95** | 95 | **95** | 95 | 95 | 95 | 95 | 95 | 95 | 95 |  |
| **264** | 95 | 95 | 80 | 82 | **87** | 87 | **84** | 84 | 95 | 93 | 87 | 83 | 85 | 95 |  |
| **638** | 95 | 95 | 85 | 95 | **93** | 95 | **95** | 93 | 93 | 95 | 95 | 95 | 93 | 95 |  |
| **642** | 95 | 95 | 93 | 92 | **95** | 94 | **82** | 94 | 84 | 94 | 95 | 93 | 93 | 85 |  |
| ***Mean*** | 78.75 | 77.37 | 64.75 | 72.5 | **68.75** | 69.75 | **68.75** | 73.75 | 71.37 | 69.5 | 73.37 | 68.5 | 76.25 | 76.12 |  |
| Q1 | 66.25 | 60.5 | 45.75 | 53 | **47.5** | 42.5 | **50.75** | 52 | 49 | 46 | 49.25 | 42.75 | 59.25 | 59.25 |  |
| Q3 | 95 | 95 | 87 | 92.75 | **93.5** | 94.25 | **86.75** | 93.25 | 93.5 | 94.25 | 95 | 93.5 | 93 | 95 |  |
| **IQR** | 28.75 | 34.5 | 41.25 | 39.75 | **46** | 51.75 | **36** | 41.25 | 44.5 | 48.25 | 45.75 | 50.75 | 33.75 | 35.75 |  |
|  |  |  |  |  |  |  |  |  |  |  |  |  |  |  |  |
| **203** | 65 | 75 | 45 | 48 | 35 | **45** | **47** | 50 | 41 | 43 | 43 | 40 | 45 | 50 | **GR10CS+9.5CS-** |
| **210** | 53 | 43 | 23 | 35 | 30 | **30** | **35** | 35 | 25 | 33 | 38 | 25 | 45 | 45 |  |
| **256** | 83 | 82 | 70 | 60 | 65 | **59** | **66** | 67 | 50 | 70 | 71 | 68 | 69 | 66 |  |
| **261** | 95 | 79 | 76 | 66 | 68 | **67** | **67** | 68 | 66 | 67 | 68 | 63 | 60 | 81 |  |
| **263** | 68 | 67 | 60 | 60 | 54 | **54** | **55** | 53 | 43 | 47 | 58 | 47 | 48 | 50 |  |
| **265** | 95 | 91 | 69 | 71 | 63 | **63** | **69** | 69 | 65 | 70 | 62 | 74 | 67 | 77 |  |
| **639** | 63 | 42 | 45 | 47 | 50 | **52** | **47** | 47 | 43 | 45 | 47 | 42 | 50 | 62 |  |
| **640** | 95 | 95 | 80 | 90 | 85 | **95** | **85** | 77 | 95 | 85 | 84 | 99 | 95 | 95 |  |
| **641** | 60 | 48 | 33 | 30 | 55 | **30** | **28** | 25 | 25 | 25 | 25 | 25 | 25 | 40 |  |
| ***Mean*** | 71 | 65.2 | 52.4 | 53.5 | 53 | **52.5** | **52.4** | 52.1 | 48.6 | 50.3 | 51.9 | 51.1 | 54.2 | 60.4 |  |
| Q1 | 60.75 | 44.25 | 36 | 38 | 38.75 | **33.75** | **38** | 38 | 35 | 35.5 | 39.25 | 31 | 45 | 46.25 |  |
| Q3 | 92 | 81.25 | 69.75 | 64.5 | 64.5 | **62** | **66.75** | 67.75 | 61.25 | 69.25 | 66.5 | 66.75 | 65.25 | 74.25 |  |
| **IQR** | 31.25 | 37 | 33.75 | 26.5 | 25.75 | **28.25** | **28.75** | 29.75 | 26.25 | 33.75 | 27.25 | 35.75 | 20.25 | 28 |  |
|  |  |  |  |  |  |  |  |  |  |  |  |  |  |  |  |
| **Mean** | 77.36 | 74.72 | 62 | 66.44 | 63.12 | 63.2 | **63.24** | 66.48 | 61.12 | 62.12 | 65.4 | 62.92 | 67.36 | 70.76 |  |
| **Q1** | 65 | 53 | 45 | 48 | 50 | 44 | **47** | 50 | 41 | 43 | 47 | 42 | 50 | 52 |  |
| **Q3** | 95 | 95 | 80 | 90 | 85 | 87 | **82** | 84 | 84 | 85 | 84 | 83 | 85 | 85 |  |
| **IQR** | 30 | 42 | 35 | 42 | 35 | 43 | **35** | 34 | 43 | 42 | 37 | 41 | 35 | 33 |  |

**Supplementary Table 2| Sound pressure level (SPL) for each tone frequency and each animal which elicited small but still detectable alpha startle responses.**

| **CR percentage** | | | | | | | | | | | | | | | **10kHz CS+** | | |
| --- | --- | --- | --- | --- | --- | --- | --- | --- | --- | --- | --- | --- | --- | --- | --- | --- | --- |
|  | | **Grp.10CS+4CS-( n = 8 mice)** | | | | **Grp.10CS+9CS-(n = 8 mice)** | | | | **Grp.10CS+9.5CS-(n = 8 mice)** | | | | | **Grp.10CS+4CS- vs Grp.10CS+9.5CS-** | **Grp.10CS+4CS- vs Grp.10CS+9CS-** | **Grp.10CS+9CS- vs Grp.10CS+9.5CS-** |
|  | | **10 kHz** | **4 kHz** | **p-value** | **q-value** | **10 kHz** | **9 kHz** | **p-value** | **q-value** | **10 kHz** | **9.5 kHz** | | **p-value** | **q-value** |  |  |  |
| **Session 1** | | 14.40(±6.8) | 9.37(±7.4) | 0.744 | 0.900 | 26.21(±6.8) | 18.54(±5.4) | 0.505 | 0.698 | 32.46(±8.4) | 21.00(±5.3) | | 0.443 | 0.953 | 0.3965 | 0.4757 | 0.5937 |
| **Session 2** | | 11.46(±3.9) | 7.75(±4.7) | 0.814 | 0.936 | 26.65(±7.2) | 19.58(±8.4) | 0.547 | 0.746 | 41.36(±6.9) | 27.00(±6.5) | | 0.313 | 0.944 | 0.0506 | 0.2156 | 0.2156 |
| **Session 3** | | 20.56(±8.5) | 16.75(±8.9) | 0.810 | 0.934 | 40.11(±12.9) | 30.62(±13.2) | 0.402 | 0.612 | 51.99(±8.7) | 33.68(±9.8) | | 0.178 | 0.878 | 0.0378 | 0.1569 | 0.3142 |
| **Session 4** | | 16.27(±7.8) | 11.23(±6.3) | 0.744 | 0.900 | 34.42(±12.7) | 25.89(±12.7) | 0.454 | 0.663 | 36.32(±9.2) | 29.86(±10.5) | | 0.674 | 0.984 | 0.1951 | 0.1951 | 0.8707 |
| **Session 5** | | 19.45(±8.6) | 16.62(±9.3) | 0.863 | 0.955 | 40.91(±11.4) | 30.07(±8.9) | 0.333 | 0.558 | 40.72(±8.3) | 26.48(±7.4) | | 0.313 | 0.944 | 0.1173 | 0.1173 | 0.9874 |
| **Session 6** | | 27.06(±13.5) | 19.17(±9.8) | 0.577 | 0.778 | 34.25(±11.3) | 24.89(±9.6) | 0.404 | 0.612 | 45.34(±8.9) | 37.24(±10.5) | | 0.588 | 0.966 | 0.3824 | 0.5395 | 0.5197 |
| **Session 7** | | 37.88(±15.5) | 25.09(±13.2) | 0.331 | 0.551 | 45.46(±12.6) | 38.98(±12.3) | 0.584 | 0.771 | 48.32(±11.7) | 37.25(±11.2) | | 0.455 | 0.953 | 0.7769 | 0.7769 | 0.8060 |
| **Session 8** | | 36.38(±10.6) | 26.11(±10.5) | 0.455 | 0.688 | 41.88(±12.0) | 35.38(±12.5) | 0.577 | 0.767 | 45.41(±12.9) | 34.20(±10.1) | | 0.451 | 0.953 | 0.7624 | 0.7624 | 0.7624 |
| **Session 9** | | 46.84(±12.6) | 28.26(±12.8) | 0.131 | 0.276 | 51.27(±13.7) | 43.64(±13.3) | 0.506 | 0.698 | 46.72(±13.7) | 37.72(±14.5) | | 0.539 | 0.953 | 0.9918 | 0.9918 | 0.9918 |
| **Session 10** | | 56.25(±10.2) | 36.15(±12.5) | 0.101 | 0.235 | 53.94(±12.8) | 44.41(±13.2) | 0.401 | 0.612 | 51.51(±11.1) | 36.00(±11.0) | | 0.275 | 0.944 | 0.8430 | 0.8430 | 0.8430 |
| **Session 11** | | 54.31(±9.1) | 32.11(±10.0) | 0.067 | 0.175 | 49.62(±12.9) | 44.37(±13.7) | 0.662 | 0.822 | 42.04(±11.4) | 32.33(±11.5) | | 0.516 | 0.953 | 0.6880 | 0.6880 | 0.6880 |
| **Session 12** | | 55.07(±14.0) | 35.22(±10.9) | 0.106 | 0.242 | 59.82(±11.6) | 44.74(±12.0) | 0.159 | 0.343 | 48.50(±12.2) | 35.08(±13.2) | | 0.377 | 0.953 | 0.6309 | 0.6842 | 0.6309 |
| **Session 13** | | 56.07(±13.2) | 27.09(±7.5) | 0.015 | 0.060 | 59.28(±12.6) | 47.98(±9.5) | 0.310 | 0.531 | 58.77(±12.4) | 40.59(±12.5) | | 0.214 | *0.941* | 0.9715 | 0.9715 | 0.9715 |
| **Session 14** | | 60.39(±11.6) | 28.97(±7.8) | 0.008 | 0.036 | 59.93(±12.2) | 46.43(±15.4) | 0.206 | 0.394 | 59.52(±10.3) | 49.72(±13.3) | | 0.569 | 0.966 | 0.9685 | 0.9685 | 0.9685 |
| **Session 15** | | 59.20(±13.8) | 29.88(±11.8) | 0.014 | 0.057 | 61.97(±13.27) | 46.00(±12.5) | 0.136 | 0.318 | 60.97(±10.0) | 49.26(±11.4) | | 0.492 | 0.953 | 0.9425 | 0.9425 | 0.9425 |
| **Session 16** | | 54.68(±13.9) | 28.18(±12.9) | 0.026 | 0.086 | 64.09(±14.9) | 51.43(±14.6) | 0.245 | 0.451 | 64.54(±9.2) | 48.62(±14.7) | | 0.328 | 0.944 | 0.8395 | 0.8395 | 0.8395 |
| **Session 17** | | 60.96(±12.5) | 32.76(±11.1) | 0.018 | 0.069 | 68.65(±10.6) | 56.35(±11.6) | 0.262 | 0.474 | 66.00(±10.6) | 52.06(±12.0) | | 0.404 | 0.953 | 0.8460 | 0.8460 | 0.8460 |
| **Session 18** | | 70.29(±10.0) | 33.13(±13.2) | 0.001 | 0.009 | 65.64(±13.5) | 56.26(±13.8) | 0.404 | 0.612 | 67.08(±11.3) | 51.25(±13.1) | | 0.331 | 0.944 | 0.9220 | 0.9220 | 0.9220 |
| Session | | F(17,245)=7.87,p<.0001 | | | | F(17,245)=8.94,p<.0001 | | | | F(17,221)=2.90,p=0.0002 | | | | |  | | |
| 10 kHz | F(17,119)=6.65, p<.0001 | | | | | 10 kHz | F(17,119)=5.05,p<.0001 | | | 10 kHz | | F(17,107)=1.96,p=0.019 | | | Group: F(2,21)=0.713,p=0.501 | | |
| 4 kHz | F(17,119)=2.62,p=0.0012 | | | | | 9 kHz | F(17,119)=3.93,p<.0001 | | | 9.5 kHz | | F(17,107)=1.33,p=0.186 | | | Session:F(17,345)=12.33, p<.0001 | | |
| Trial | | F(1,245)=55.49,p<.0001 | | | | F(1,245)=25.04,p<.0001 | | | | F(1,221)=34.91,p<.0001 | | | | | Session*Group:F(34,345)=1.01,p=0.324 | | |
| Session*Trial | | *F(17,245)=1.28,p=0.20* | | | | *F(17,245)=0.13,p=1.00* | | | | *F(17,221)=0.14,p=1.00* | | | | |  | | |

| **Eye closure – all trials** | | | | | | | | | | | | | | | | **10kHz CS+** | | |
| --- | --- | --- | --- | --- | --- | --- | --- | --- | --- | --- | --- | --- | --- | --- | --- | --- | --- | --- |
|  | | **Grp.10CS+4CS-( n = 8 mice)** | | | | **Grp.10CS+9CS-(n = 8 mice)** | | | | | **Grp.10CS+9.5CS-(n = 8 mice)** | | | | | **Grp.10CS+4CS- vs Grp.10CS+9.5CS-** | **Grp.10CS+4CS- vs Grp.10CS+9CS-** | **Grp.10CS+9CS- vs Grp.10CS+9.5CS-** |
|  | | **10 kHz** | **4 kHz** | **p-value** | **q-value** | **10 kHz** | | **9 kHz** | **p-value** | **q-value** | **10 kHz** | | **9.5 kHz** | **p-value** | **q-value** |  |  |  |
| **Session 1** | | 0.04(±0.01) | 0.02(±0.01) | 0.999 | 1.00 | 0.10(±0.03) | | 0.06(±0.02) | 0.750 | 0.905 | 0.09(±0.03) | | 0.04(±0.01) | 0.048 | 0.070 | 0.7397 | 0.7397 | 0.9045 |
| **Session 2** | | 0.04(±0.01) | 0.03(±0.01) | 0.999 | 1.00 | 0.06(±0.02) | | 0.05(±0.01) | 0.999 | 1.00 | 0.13(±0.04) | | 0.05(±0.009) | <.0001 | <.0001 | 0.3991 | 0.8049 | 0.3991 |
| **Session 3** | | 0.06(±0.02) | 0.04(±0.01) | 0.929 | 1.00 | 0.12(±0.04) | | 0.08(±0.02) | 0.0690 | 0.096 | 0.22(±0.09) | | 0.10(±0.04) | <.0001 | <.0001 | 0.0841 | 0.4980 | 0.1647 |
| **Session 4** | | 0.05(±0.02) | 0.02(±0.01) | 0.960 | 1.00 | 0.09(±0.04) | | 0.05(±0.02) | 0.661 | 0.807 | 0.17(±0.07) | | 0.06(±0.02) | <.0001 | <.0001 | 0.2783 | 0.6454 | 0.3148 |
| **Session 5** | | 0.06(±0.03) | 0.04(±0.02) | 0.963 | 1.00 | 0.16(±0.05) | | 0.08(±0.02) | <.0001 | <.0001 | 0.15(±0.07) | | 0.07(±0.03) | <.0001 | <.0001 | 0.3131 | 0.3131 | 0.9193 |
| **Session 6** | | 0.08(±0.04) | 0.05(±0.02) | 0.616 | 1.00 | 0.08(±0.02) | | 0.05(±0.01) | 0.776 | 0.930 | 0.16(±0.07) | | 0.10(±0.04) | <.0001 | <.0001 | 0.3215 | 0.9961 | 0.3215 |
| **Session 7** | | 0.15(±0.05) | 0.08(±0.03) | <.0001 | <.0001 | 0.15(±0.05) | | 0.09(±0.02) | 0.005 | 0.007 | 0.19(±0.06) | | 0.08(±0.02) | <.0001 | <.0001 | 0.7008 | 0.8621 | 0.7008 |
| **Session 8** | | 0.15(±0.05) | 0.07(±0.03) | <.0001 | <.0001 | 0.16(±0.05) | | 0.10(±0.04) | <.0001 | <.0001 | 0.20(±0.08) | | 0.09(±0.02) | <.0001 | <.0001 | 0.7607 | 0.7607 | 0.7607 |
| **Session 9** | | 0.18(±0.05) | 0.08(±0.03) | <.0001 | <.0001 | 0.24(±0.11) | | 0.17(±0.08) | <.0001 | <.0001 | 0.20(±0.09) | | 0.10(±0.04) | <.0001 | <.0001 | 0.7769 | 0.7769 | 0.7769 |
| **Session 10** | | 0.29(±0.11) | 0.10(±0.04) | <.0001 | <.0001 | 0.29(±0.11) | | 0.17(±0.07) | <.0001 | <.0001 | 0.18(±0.05) | | 0.09(±0.03) | <.0001 | <.0001 | 0.1635 | 0.8769 | 0.1635 |
| **Session 11** | | 0.28(±0.09) | 0.10(±0.03) | <.0001 | <.0001 | 0.27(±0.10) | | 0.17(±0.08) | <.0001 | <.0001 | 0.17(±0.06) | | 0.10(±0.04) | <.0001 | <.0001 | 0.1893 | 0.8639 | 0.1893 |
| **Session 12** | | 0.31(±0.12) | 0.11(±0.03) | <.0001 | <.0001 | 0.30(±0.10) | | 0.17(±0.07) | <.0001 | <.0001 | 0.22(±0.08) | | 0.11(±0.05) | <.0001 | <.0001 | 0.1795 | 0.8764 | 0.1795 |
| **Session 13** | | 0.31(±0.12) | 0.08(±0.02) | <.0001 | <.0001 | 0.30(±0.09) | | 0.16(±0.06) | <.0001 | <.0001 | 0.36(±0.12) | | 0.13(±0.05) | <.0001 | <.0001 | 0.7998 | 0.7998 | 0.7998 |
| **Session 14** | | 0.36(±0.10) | 0.09(±0.02) | <.0001 | <.0001 | 0.35(±0.13) | | 0.19(±0.09) | <.0001 | <.0001 | 0.31(±0.09) | | 0.17(±0.06) | <.0001 | <.0001 | 0.6475 | 0.7892 | 0.6475 |
| **Session 15** | | 0.35(±0.11) | 0.10(±0.04) | <.0001 | <.0001 | 0.33(±0.12) | | 0.18(±0.06) | <.0001 | <.0001 | 0.36(±0.11) | | 0.19(±0.05) | <.0001 | <.0001 | 0.9227 | 0.9227 | 0.9227 |
| **Session 16** | | 0.30(±0.11) | 0.11(±0.05) | <.0001 | <.0001 | 0.36(±0.11) | | 0.20(±0.07) | <.0001 | <.0001 | 0.33(±0.09) | | 0.18(±0.06) | <.0001 | <.0001 | 0.7516 | 0.7516 | 0.7516 |
| **Session 17** | | 0.40(±0.10) | 0.12(±0.04) | <.0001 | <.0001 | 0.42(±0.12) | | 0.21(±0.06) | <.0001 | <.0001 | 0.37(±0.13) | | 0.16(±0.04) | <.0001 | <.0001 | 0.6359 | 0.6359 | 0.6359 |
| **Session 18** | | 0.46(±0.10) | 0.14(±0.05) | <.0001 | <.0001 | 0.45(±0.15) | | 0.24(±0.08) | <.0001 | <.0001 | 0.43(±0.13) | | 0.18(±0.06) | <.0001 | <.0001 | 0.8822 | 0.8822 | 0.8822 |
| Session | | F(17,27551)=267.06,p<.0001 | | | | F(17,26362)=286.92,p<.0001 | | | | | F(17,24822)=127.76,p<.0001 | | | | |  | | |
| 10 kHz | F(17,13811)=227.52,p<.0001 | | | | | 10 kHz | F(17,13277)=188.30, p<.0001 | | | | 10 kHz | F(17,12459)=88.90,p<.0001 | | | | Group: F(2,21)=0.050, p=0.951 | | |
| 4 kHz | F(17,27569)=227.59,p<.0001 | | | | | 9 kHz | F(17,13128)=106.20,p<.0001 | | | | 9.5 kHz | F(17,12356)=51.38,p>.0001 | | | | Session: F(17,39497)=464.27,p<.0001 | | |
| Trial | | F(17,27551)=3011.13,p<.0001 | | | | F(1,26362)=1292.91,p<.0001 | | | | | F(1,24822)=1883.90,p<.0001 | | | | | Group*Session: F(34,34497)=21.06,p<.0001 | | |
| Session*Trial | | F(17,27551)=105.21,p<.0001 | | | | F(17,26362)=29.53,p<.0001 | | | | | F(17,24822)=23.97,p<.0001 | | | | |  | | |

| **Eye closure – CR only trials** | | | | | | | | | | | | | | | | **10kHz CS+** | | |
| --- | --- | --- | --- | --- | --- | --- | --- | --- | --- | --- | --- | --- | --- | --- | --- | --- | --- | --- |
|  | | **Grp.10CS+4CS-( n = 8 mice)** | | | | **Grp.10CS+9CS-(n = 8 mice)** | | | | | **Grp.10CS+9.5CS-(n = 8 mice)** | | | | | **Grp.10CS+4CS- vs Grp.10CS+9.5CS-** | **Grp.10CS+4CS- vs Grp.10CS+9CS-** | **Grp.10CS+9CS- vs Grp.10CS+9.5CS-** |
|  | | **10 kHz** | **4 kHz** | **p-value** | **q-value** | **10 kHz** | **9 kHz** | | **p-value** | **q-value** | **10 kHz** | | **9.5 kHz** | **p-value** | **q-value** |  |  |  |
| **Session 1** | | 0.12(±0.02) | 0.10(±0.02) | 0.308 | 0.345 | 0.23(±0.08) | 0.15(±0.03) | | 0.999 | 1.00 | 0.19(±0.04) | | 0.10(±0.01) | 0.138 | 0.191 | 0.9873 | 0.8827 | 0.8827 |
| **Session 2** | | 0.14(±0.03) | 0.14(±0.03) | 0.399 | 0.437 | 0.13(±0.02) | 0.11(±0.01) | | 0.999 | 1.00 | 0.22(±0.04) | | 0.12(±0.01) | 0.0003 | 0.0005 | 0.5035 | 0.9053 | 0.5035 |
| **Session 3** | | 0.17(±0.04) | 0.19(±0.10) | 0.109 | 0.130 | 0.21(±0.04) | 0.18(±0.03) | | 0.477 | 0.623 | 0.31(±0.10) | | 0.16(±0.04) | <.0001 | <.0001 | 0.2637 | 0.7643 | 0.2637 |
| **Session 4** | | 0.15(±0.04) | 0.12(±0.02) | 0.012 | 0.016 | 0.20(±0.05) | 0.16(±0.03) | | 0.878 | 1.00 | 0.39(±0.20) | | 0.16(±0.03) | <.0001 | <.0001 | 0.0795 | 0.7700 | 0.0795 |
| **Session 5** | | 0.16(±0.04) | 0.16(±0.04) | 0.256 | 0.331 | 0.31(±0.06) | 0.20(±0.03) | | <.0001 | <.0001 | 0.28(±0.10) | | 0.18(±0.05) | <.0001 | <.0001 | 0.7968 | 0.7968 | 0.9228 |
| **Session 6** | | 0.22(±0.06) | 0.19(±0.05) | 0.002 | 0.0040 | 0.19(±0.03) | 0.14(±0.02) | | 0.861 | 1.00 | 0.28(±0.08) | | 0.20(±0.04) | 0.0004 | 0.0006 | 0.9505 | 0.2044 | 0.2044 |
| **Session 7** | | 0.26(±0.07) | 0.18(±0.03) | <.0001 | <.0001 | 0.24(±0.04) | 0.18(±0.04) | | 0.0007 | 0.001 | 0.30(±0.07) | | 0.16(±0.02) | <.0001 | <.0001 | 0.7584 | 0.5060 | 0.5060 |
| **Session 8** | | 0.25(±0.07) | 0.20(±0.06) | <.0001 | <.0001 | 0.28(±0.06) | 0.20(±0.04) | | <.0001 | <.0001 | 0.39(±0.10) | | 0.19(±0.04) | <.0001 | <.0001 | 0.6017 | 0.6017 | 0.6017 |
| **Session 9** | | 0.30(±0.06) | 0.16(±0.03) | <.0001 | <.0001 | 0.35(±0.11) | 0.27(±0.09) | | 0.0002 | 0.0004 | 0.34(±0.09) | | 0.20(±0.04) | <.0001 | <.0001 | 0.8423 | 0.8423 | 0.8671 |
| **Session 10** | | 0.41(±0.12) | 0.20(±0.04) | <.0001 | <.0001 | 0.41(±0.10) | 0.30(±0.07) | | <.0001 | <.0001 | 0.30(±0.07) | | 0.19(±0.04) | <.0001 | <.0001 | 0.2423 | 0.6762 | 0.2599 |
| **Session 11** | | 0.44(±0.12) | 0.23(±0.04) | <.0001 | <.0001 | 0.42(±0.11) | 0.28(±0.08) | | <.0001 | <.0001 | 0.39(±0.08) | | 0.25(±0.04) | <.0001 | <.0001 | 0.4997 | 0.8553 | 0.4997 |
| **Session 12** | | 0.48(±0.10) | 0.26(±0.05) | <.0001 | <.0001 | 0.42(±0.10) | 0.27(±0.07) | | <.0001 | <.0001 | 0.37(±0.08) | | 0.23(±0.05) | <.0001 | <.0001 | 0.3014 | 0.4621 | 0.4621 |
| **Session 13** | | 0.49(±0.10) | 0.25(±0.03) | <.0001 | <.0001 | 0.43(±0.09) | 0.27(±0.06) | | <.0001 | <.0001 | 0.52(±0.11) | | 0.29(±0.04) | <.0001 | <.0001 | 0.5346 | 0.5346 | 0.5157 |
| **Session 14** | | 0.55(±0.07) | 0.29(±0.05) | <.0001 | <.0001 | 0.47(±0.12) | 0.30(±0.08) | | <.0001 | <.0001 | 0.46(±0.11) | | 0.28(±0.06) | <.0001 | <.0001 | 0.7030 | 0.7030 | 0.8957 |
| **Session 15** | | 0.51(±0.09) | 0.29(±0.05) | <.0001 | <.0001 | 0.45(±0.10) | 0.30(±0.06) | | <.0001 | <.0001 | 0.52(±0.10) | | 0.35(±0.03) | <.0001 | <.0001 | 0.8164 | 0.5652 | 0.5652 |
| **Session 16** | | 0.46(±0.11) | 0.28(±0.05) | <.0001 | <.0001 | 0.47(±0.10) | 0.31(±0.07) | | <.0001 | <.0001 | 0.49(±0.10) | | 0.31(±0.05) | <.0001 | <.0001 | 0.9480 | 0.9480 | 0.9480 |
| **Session 17** | | 0.57(±0.08) | 0.30(±0.03) | <.0001 | <.0001 | 0.53(±0.11) | 0.31(±0.07) | | <.0001 | <.0001 | 0.48(±0.11) | | 0.26(±0.03) | <.0001 | <.0001 | 0.7061 | 0.7061 | 0.7061 |
| **Session 18** | | 0.61(±0.07) | 0.31(±0.05) | <.0001 | <.0001 | 0.58(±0.14) | 0.36(±0.08) | | <.0001 | <.0001 | 0.56(±0.10) | | 0.29(±0.05) | <.0001 | <.0001 | 0.9580 | 0.9580 | 0.9580 |
|  | |  | |  | |  | | | | |  | | | | |  | | |
| Session | | F(17,9026)=99.45,p<.0001 | | | | F(17,11653)=144.18,p<.0001 | | | | | F(17,10708)=105.81,p<.0001 | | | | |  | | |
| 10kHz | F(17,5766)=73.54,p<.0001 | | | | | 10 kHz | | F(17,6479)=103.97,p<.0001 | | | 10 kHz | F(17,6148)=77.56,p<.0001 | | | | Group: F(1,21)=0.44,p=0.645 | | |
| 4 kHz | F(17,9044)=87.97,p<.0001 | | | | | 9 kHz | | F(17,5167)=45.17,p<.0001 | | | 9.5 kHz | F(17,4553)=42.69,p<.0001 | | | | Session: F(17,18393)=230.78,p<.0001 | | |
| Trial | | F(1,9026)=984.17,p<.0001 | | | | F(1,11653)=1192.1423,p<.0001 | | | | | F(110708)=1606.12,p<.0001 | | | | | Group*Session: F(34,18393)=12.26,p<.0001 | | |
| Session*Trial | | F(17,9026)=12.59,p<.0001 | | | | F(17,11653)=13.03,p<.0001 | | | | | F(17,10708) =13.59, p<.0001 | | | | |  | | |

**Supplementary Table 3| Eyeblink conditioning outcome measures during differential training (session 1-18) in response to CS+ and CS- for each group.** All values represent mean ±95%CI. The ANOVA on the linear mixed effect model (LME) for each group reveals the main effect of session in response to CS+. Abbreviation: CR, conditioned response, CS+, reinforced stimulus, CS- non reinforced stimulus.

| **CR percentage** | | | | | | | | | | **CS- comparison between groups** | | | | | | |
| --- | --- | --- | --- | --- | --- | --- | --- | --- | --- | --- | --- | --- | --- | --- | --- | --- |
|  | **Grp.10CS+4CS- ( n = 8 mice)** | | | **Grp.10CS+9CS- (n = 8 mice)** | | | **Grp.10CS+9.5CS- (n =6 mice)** | | | | **Grp.10CS+4CS- vs Grp.10CS+9.5CS-** | | **Grp.10CS+4CS- vs Grp.10CS+9CS-** | | **Grp.10CS+9.5CS- vs.GRP.10CS+9CS-** | |
| **CS-only** | **CS-** | **p-value** | **q-value** | **CS-** | **p-value** | **q-value** | **CS-** | **p-value** | **q-value** | | **p-value** | **q-value** | **p-value** | **q-value** | **p-value** | **q-value** |
| 2 kHz | 30.39(±18.2) | 0.0006 | 0.027 | 61.83(±14.9) | 0.999 | 1.00 | 38.80(±19.7) | 0.818 | 1.00 | | 0.249 | 0.999 | 0.042 | 0.999 | 0.718 | 0.999 |
| 4 kHz | 49.34(±14.0) | **0.344** | **1.00** | 66.13(±15.0) | 1.00 | 1.00 | 53.50(±19.3) | 1.00 | 1.00 | | 0.261 | 0.999 | 0.359 | 0.999 | 0.944 | 0.999 |
| 6 kHz | 36.99(±15.7) | 0.008 | 0.100 | 53.30(±17.7) | 0.604 | 1.00 | 35.05(±16.6) | 0.467 | 1.00 | | 0.735 | 0.999 | 0.379 | 0.999 | 0.868 | 0.999 |
| 8 kHz | 61.62(±15.2) | 0.993 | 1.00 | 72.81(±14.0) | 0.998 | 1.00 | 49.98(±22.0) | 1.00 | 1.00 | | 0.952 | 0.999 | 0.625 | 0.999 | 0.837 | 0.999 |
| 9 kHz | 56.10(±15.0) | 0.843 | 1.00 | 67.27(±12.6) | **1.00** | **1.00** | 49.69(±20.5) | 1.00 | 1.00 | | 0.763 | 0.999 | 0.626 | 0.999 | 0.986 | 0.999 |
| 9.5 kHz | 68.32(±11.3) | 0.999 | 1.00 | 72.60(±20.0) | 0.999 | 1.00 | 49.30(±20.4) | **1.00** | **1.00** | | 0.978 | 0.999 | 0.932 | 0.999 | 0.857 | 0.999 |
| ***10 kHz*** | **72.60(**±**12.0)** | ***1.00*** | **1.00** | **67.55(**±**12.8)** | ***1.00*** | **1.00** | **52.12(**±**20.2)** | ***1.00*** | **1.00** | | ***0.968*** | ***0.999*** | ***0.907*** | ***0.999*** | ***0.987*** | ***0.999*** |
| 10.5 kHz | 71.22(±8.8) | 1.00 | 1.00 | 74.44(±14.8) | 0.998 | 1.00 | 45.81(±18.8) | 0.008 | 1.00 | | 0.668 | 0.999 | 0.961 | 0.999 | 0.517 | 0.999 |
| 11 kHz | 61.16(±11.8) | 0.990 | 1.00 | 75.07(±9.5) | 0.995 | 1.00 | 39.61(±18.6) | 0.875 | 1.00 | | 0.797 | 0.999 | 0.489 | 0.999 | 0.223 | 0.999 |
| 12 kHz | 60.73(±12.6) | 0.986 | 1.00 | 74.31(±16.1) | 0.998 | 1.00 | 43.28(±18.3) | 0.981 | 1.00 | | 0.946 | 0.999 | 0.505 | 0.999 | 0.378 | 0.999 |
| 14 kHz | 74.47(±7.6) | 1.00 | 1.00 | 72.40(±12.7) | 0.999 | 1.00 | 47.40(±18.7) | 0.999 | 1.00 | | 0.663 | 0.999 | 0.983 | 0.999 | 0.759 | 0.999 |
| 16 kHz | 67.03(±9.5) | 0.999 | 1.00 | 67.35(±19.4) | 1.00 | 1.00 | 44.38(±19.8) | 0.997 | 1.00 | | 0.817 | 0.999 | 0.999 | 0.999 | 0.804 | 0.999 |
| 18 kHz | 61.83(±14.7) | 0.994 | 1.00 | 72.52(±12.2) | 0.999 | 1.00 | 47.89(±19.7) | 0.999 | 1.00 | | 0.997 | 0.999 | 0.650 | 0.999 | 0.733 | 0.999 |
| 20 kHz | 67.66(±13.8) | 0.999 | 1.00 | 67.39(±12.4) | 1.00 | 1.00 | 41.41(±15.5) | 0.926 | 1.00 | | 0.556 | 0.999 | 0.999 | 0.999 | 0.569 | 0.999 |
| *Tone Frequency:* | *F(13,91)=4.31, p<.0001* | | | *F(13,91)=1.75, p=0.063* | | | *F(13,65)=1.13, p=0.346* | | | | *Tone Frequency: F(13,247)=5.22,p<.0001* | | | | | |
|  |  | | |  | | |  | | | | *Group: F(2,19)=0.576,p=0.571* | | | | | |
|  |  | | |  | | |  | | | | *Tone Frequency*Group:F(26,247)=1.35,p=0.123* | | | | | |
|  |  | | |  | | |  | | | |  | | | | | |

| **Eye closure – all trials** | | | | | | | | | | **CS- comparison between groups** | | | | | | |
| --- | --- | --- | --- | --- | --- | --- | --- | --- | --- | --- | --- | --- | --- | --- | --- | --- |
|  | **Grp.10CS+4CS- ( n = 8 mice)** | | | **Grp.10CS+9CS- (n = 8 mice)** | | | **Grp.10CS+9.5CS- (n =6 mice)** | | | **Grp.10CS+4CS- vs Grp.10CS+9.5CS-** | | **Grp.10CS+4CS- vs Grp.10CS+9CS-** | | | **Grp.10CS+9.5CS- vs.GRP.10CS+9CS-** | |
| **CS-only** | **CS-** | **p-value** | **q-value** | **CS-** | **p-value** | **q-value** | **CS-** | **p-value** | **q-value** | **p-value** | **q-value** | | **p-value** | **q-value** | **p-value** | **q-value** |
| 2 kHz | 0.12(±0.07) | <.0001 | <.0001 | 0.30(±0.12) | <.0001 | 0.001 | 0.23(±0.08) | 0.0003 | 0.033 | 0.555 | 0.999 | | 0.208 | 0.999 | 0.830 | 0.999 |
| 4 kHz | **0.21(±0.07)** | **<.0001** | **<.0001** | 0.37(±0.16) | 0.198 | 0.667 | 0.38(±0.11) | 0.996 | 1.00 | 0.302 | 0.999 | | 0.297 | 0.999 | 0.994 | 0.999 |
| 6 kHz | 0.21(±0.11) | <.0001 | <.0001 | 0.30(±0.14) | 0.0001 | 0.001 | 0.25(±0.09) | 0.002 | 0.087 | 0.955 | 0.999 | | 0.711 | 0.999 | 0.895 | 0.999 |
| 8 kHz | 0.32(±0.12) | <.0001 | <.0001 | 0.41(±0.15) | 0.877 | 1.00 | 0.37(±0.14) | 0.985 | 1.00 | 0.823 | 0.999 | | 0.599 | 0.999 | 0.948 | 0.999 |
| 9 kHz | 0.30(±0.10) | <.0001 | <.0001 | **0.36(±0.13)** | **0.003** | **0.032** | 0.30(±0.08) | 0.013 | 0.280 | 0.935 | 0.999 | | 0.872 | 0.999 | 0.698 | 0.999 |
| 9.5 kHz | 0.45(±0.10) | 0.591 | 0.980 | 0.50(±0.20) | 0.999 | 1.00 | **0.38(±0.13)** | **0.987** | **1.00** | 0.811 | 0.999 | | 0.872 | 0.999 | 0.530 | 0.999 |
| ***10 kHz*** | **0.53(±0.16)** | ***1.00*** | ***1.00*** | **0.46(±0.17)** | ***1.00*** | ***1.00*** | **0.43(±0.12)** | ***1.00*** | **1.00** | ***0.613*** | ***0.999*** | | ***0.787*** | ***0.999*** | ***0.939*** | ***0.999*** |
| 10.5 kHz | 0.45(±0.09) | 0.616 | 0.980 | 0.45(±0.15) | 0.999 | 1.00 | 0.37(±0.12) | 0.994 | 1.00 | 0.785 | 0.999 | | 0.999 | 0.999 | 0.804 | 0.999 |
| 11 kHz | 0.35(±0.09) | <.0001 | 0.0002 | 0.40(±0.12) | 0.695 | 1.00 | 0.34(±0.11) | 0.686 | 1.00 | 0.997 | 0.999 | | 0.881 | 0.999 | 0.867 | 0.999 |
| 12 kHz | 0.38(±0.13) | 0.007 | 0.020 | 0.42(±0.17) | 0.999 | 1.00 | 0.35(±0.11) | 0.893 | 1.00 | 0.950 | 0.999 | | 0.893 | 0.999 | 0.754 | 0.999 |
| 14 kHz | 0.43(±0.08) | 0.248 | 0.481 | 0.45(±0.17) | 0.999 | 1.00 | 0.36(±0.09) | 0.915 | 1.00 | 0.787 | 0.999 | | 0.957 | 0.999 | 0.634 | 0.999 |
| 16 kHz | 0.38(±0.10) | 0.007 | 0.020 | 0.40(±0.17) | 0.846 | 1.00 | 0.38(±0.13) | 0.997 | 1.00 | 0.998 | 0.999 | | 0.985 | 0.999 | 0.975 | 0.999 |
| 18 kHz | 0.41(±0.14) | 0.406 | 0.726 | 0.43(±0.14) | 0.993 | 1.00 | 0.35(±0.12) | 0.916 | 1.00 | 0.744 | 0.999 | | 0.990 | 0.999 | 0.812 | 0.999 |
| 20 kHz | 0.38(±0.11) | 0.002 | 0.007 | 0.39(±0.16) | 0.574 | 1.00 | 0.32(±0.07) | 0.399 | 1.00 | 0.878 | 0.999 | | 0.991 | 0.999 | 0.820 | 0.999 |
| *Tone Frequency* | *F(13,1841)=21.36,p<.0001* | | | *F(13,1737)=6.78, p<.0001* | | | *F(13,1375)=3.64, p<.0001* | | | *Tone Frequency:* *F(13,4953)=26.68,p<.0001* | | | | | | |
|  |  | | |  | | |  | | | *Group:**F(2,19)=0.21,p=0.805* | | | | | | |
|  |  | | |  | | |  | | | *Tone Frequency*Group:* *F(26,4953)=3.48,p<.0001* | | | | | | |

| **Eye closure – CR only trials** | | | | | | | | | | **CS- comparison between groups** | | | | | |
| --- | --- | --- | --- | --- | --- | --- | --- | --- | --- | --- | --- | --- | --- | --- | --- |
|  | **Grp.10CS+4CS- ( n = 8 mice)** | | | **Grp.10CS+9CS- (n = 8 mice)** | | | **Grp.10CS+9.5CS- (n =6 mice)** | | | **Grp.10CS+4CS- vs Grp.10CS+9.5CS-** | | **Grp.10CS+4CS- vs Grp.10CS+9CS-** | | **Grp.10CS+9.5CS- vs.GRP.10CS+9CS-** | |
| **CS-only** | **CS-** | **p-value** | **q-value** | **CS-** | **p-value** | **q-value** | **CS-** | **p-value** | **q-value** | **p-value** | **q-value** | **p-value** | **q-value** | **p-value** | **q-value** |
| 2 kHz | 0.29(±0.05) | <.0001 | <.0001 | 0.40(±0.13) | 0.001 | 0.043 | 0.41(±0.06) | 0.029 | 1.00 | 0.482 | 0.999 | 0.477 | 0.999 | 0.996 | 0.999 |
| 4 kHz | **0.41(±0.08)** | **<.0001** | **<.0001** | 0.47(±0.14) | 0.393 | 1.00 | 0.49(±0.10) | 0.833 | 1.00 | 0.792 | 0.999 | 0.887 | 0.999 | 0.975 | 0.999 |
| 6 kHz | 0.36(±0.12) | 0.001 | 0.006 | 0.45(±0.13) | 0.114 | 1.00 | 0.42(±0.09) | 0.093 | 1.00 | 0.945 | 0.999 | 0.985 | 0.999 | 0.984 | 0.999 |
| 8 kHz | 0.42(±0.12) | 0.0002 | 0.001 | 0.49(±0.15) | 0.847 | 1.00 | 0.52(±0.09) | 0.980 | 1.00 | 0.942 | 0.999 | 0.976 | 0.999 | 0.989 | 0.999 |
| 9 kHz | 0.45(±0.11) | 0.039 | 0.155 | **0.45(±0.12)** | **0.002** | **0.054** | 0.45(±0.06) | 0.203 | 1.00 | 0.739 | 0.999 | 0.665 | 0.999 | 0.999 | 0.999 |
| 9.5 kHz | 0.59(±0.08) | 0.930 | 1.00 | 0.58(±0.15) | 0.999 | 1.00 | **0.52(±0.10)** | **0.994** | **1.00** | 0.767 | 0.999 | 0.992 | 0.999 | 0.829 | 0.999 |
| ***10 kHz*** | **0.66(±0.11)** | ***1.00*** | ***1.00*** | **0.55(±0.16)** | ***1.00*** | **1.00** | **0.57(±0.09)** | ***1.00*** | **1.00** | ***0.648*** | ***0.999*** | ***0.557*** | ***0.999*** | ***0.996*** | ***0.999*** |
| 10.5 kHz | 0.60(±0.10) | 0.929 | 1.00 | 0.53(±0.12) | 0.996 | 1.00 | 0.54(±0.11) | 1.00 | 1.00 | 0.914 | 0.999 | 0.696 | 0.999 | 0.935 | 0.999 |
| 11 kHz | 0.52(±0.10) | 0.027 | 0.121 | 0.50(±0.11) | 0.755 | 1.00 | 0.56(±0.06) | 0.999 | 1.00 | 0.997 | 0.999 | 0.938 | 0.999 | 0.926 | 0.999 |
| 12 kHz | 0.55(±0.16) | 0.847 | 1.00 | 0.49(±0.15) | 0.920 | 1.00 | 0.55(±0.10) | 1.00 | 1.00 | 0.934 | 0.999 | 0.650 | 0.999 | 0.885 | 0.999 |
| 14 kHz | 0.56(±0.09) | 0.316 | 0.757 | 0.51(±0.15) | 0.995 | 1.00 | 0.54(±0.09) | 0.999 | 1.00 | 0.937 | 0.999 | 0.888 | 0.999 | 0.996 | 0.999 |
| 16 kHz | 0.53(±0.08) | 0.093 | 0.340 | 0.48(±0.14) | 0.668 | 1.00 | 0.54(±0.09) | 0.999 | 1.00 | 0.999 | 0.999 | 0.833 | 0.999 | 0.836 | 0.999 |
| 18 kHz | 0.59(±0.12) | 0.987 | 1.00 | 0.50(±0.12) | 0.762 | 1.00 | 0.49(±0.12) | 0.980 | 1.00 | 0.615 | 0.999 | 0.460 | 0.999 | 0.980 | 0.999 |
| 20 kHz | 0.52(±0.07) | 0.011 | 0.053 | 0.49(±0.16) | 0.959 | 1.00 | 0.53(±0.06) | 0.996 | 1.00 | 0.999 | 0.999 | 0.992 | 0.999 | 0.992 | 0.999 |
| *Tone Frequency* : | *F(13,1066)=9.28,p<.0001* | | | *F(13,1228)=4.22, p<.0001* | | | *F(13,839)=2.29,p=0.005* | | | *Tone Frequency:* *F(13,3133)=12.07,p<.0001* | | | | | |
|  |  | | |  | | |  | | | *Group:* *F(2,19)=0.128,p=0.880* | | | | | |
|  |  | | |  | | |  | | | *Tone Frequency *Group:* *F(26,3133)=1.99,p=0.001* | | | | | |

|  | **CR onset** | | | | | | | | | **CS- comparison between groups** | | | | | | |
| --- | --- | --- | --- | --- | --- | --- | --- | --- | --- | --- | --- | --- | --- | --- | --- | --- |
|  | **Grp.10CS+4CS- ( n = 8 mice)** | | | **Grp.10CS+9CS- (n = 8 mice)** | | | **Grp.10CS+9.5CS- (n =6 mice)** | | | | **Grp.10CS+4CS- vs Grp.10CS+9.5CS-** | | **Grp.10CS+4CS- vs Grp.10CS+9CS-** | | **Grp.10CS+9.5CS- vs.GRP.10CS+9CS-** | |
| **CS-only** | **CS-** | **p-value** | **q-value** | **CS-** | **p-value** | **q-value** | **CS-** | **p-value** | **q-value** | | **p-value** | **q-value** | **p-value** | **q-value** | **p-value** | **q-value** |
| 2 kHz | 206.03(±23.70) | 0.353 | 1.00 | 179.21(±5.40) | 0.370 | 1.00 | 168.91(±19.02) | 1.00 | 1.00 | | 0.215 | 0.719 | 0.550 | 0.888 | 0.701 | 0.893 |
| 4 kHz | **181.48(±4.95)** | **0.992** | **1.00** | 172.56(±22.96) | 1.00 | 1.00 | 204.91(±5.87) | 0.322 | 1.00 | | 0.553 | 0.888 | 0.387 | 0.847 | 0.093 | 0.624 |
| 6 kHz | 143(±15.51) | 0.999 | 1.00 | 178.07(±6.73) | 0.842 | 1.00 | 177.25(±21.66) | 0.867 | 1.00 | | 0.329 | 0.768 | 0.585 | 0.888 | 0.836 | 0.975 |
| 8 kHz | 173.45(±0.75) | 1.00 | 1.00 | 168.43(±19.11) | 1.00 | 1.00 | 191.12(±8.79) | 0.847 | 1.00 | | 0.564 | 0.888 | 0.613 | 0.888 | 0.134 | 0.679 |
| 9 kHz | 174.28(±20.41) | 0.987 | 1.00 | **167.09(±10.56)** | **0.935** | **1.00** | 189.72(±24.31) | 0.847 | 1.00 | | 0.999 | 0.999 | 0.568 | 0.888 | 0.536 | 0.888 |
| 9.5 kHz | 170.55(±13.51) | 0.999 | 1.00 | 162.3(±9.04) | 0.999 | 1.00 | **175.99(±13.47)** | **0.929** | **1.00** | | 0.960 | 0.999 | 0.700 | 0.893 | 0.516 | 0.888 |
| ***10 kHz*** | **162.20(±10.35)** | ***1.00*** | **1.00** | **154.24(±6.96)** | ***1.00*** | **1.00** | **165.85(±14.71)** | ***0.925*** | **1.00** | | **0.986** | **0.999** | **0.677** | **0.893** | **0.804** | **0.964** |
| 10.5 kHz | 164.72(±15.51) | 0.999 | 1.00 | 150.36(±9.07) | 0.999 | 1.00 | 201.23(±19.80) | 0.330 | 1.00 | | 0.309 | 0.768 | 0.200 | 0.719 | 0.014 | 0.361 |
| 11 kHz | 182.39(±24.97) | 0.999 | 1.00 | 149(±18.24) | 0.999 | 1.00 | 186.1(±16.85) | 0.710 | 1.00 | | 0.772 | 0.953 | 0.170 | 0.715 | 0.054 | 0.572 |
| 12 kHz | 187.09(±26.19) | 0.955 | 1.00 | 152.29(±23.01) | 0.999 | 1.00 | 192.17(±17.81) | 0.197 | 1.00 | | 0.592 | 0.888 | 0.403 | 0.847 | 0.088 | 0.624 |
| 14 kHz | 163.09(±14.48) | 0.999 | 1.00 | 151.06(±13.68) | 1.00 | 1.00 | 174.13(±20.39) | 0.972 | 1.00 | | 0.972 | 0.999 | 0.306 | 0.768 | 0.229 | 0.719 |
| 16 kHz | 205.39(±15.64) | 0.005 | 0.470 | 161.7(±15.46) | 0.850 | 1.00 | 162.64(±18.82) | 1.00 | 1.00 | | 0.017 | 0.361 | 0.050 | 0.572 | 0.693 | 0.893 |
| 18 kHz | 196(±9.50) | 0.928 | 1.00 | 156.31(±17.23) | 0.999 | 1.00 | 180.15(±21.80) | 0.708 | 1.00 | | 0.988 | 0.999 | 0.320 | 0.768 | 0.239 | 0.719 |
| 20 kHz | 161.67(±13.79) | 0.999 | 1.00 | 164.46(±15.51) | 0.926 | 1.00 | 201.82(±18.07) | 0.051 | 1.00 | | 0.145 | 0.679 | 0.994 | 0.999 | 0.101 | 0.624 |
| *Tone Frequency* : | *F(13, 209)=2.23,p=0.009* | | | *F(13,261)=1.99,p=0.022* | | | *F(13,243)=2.33, p=0.006* | | | | *Tone Frequency: F(13,713)=2.28,p=0.005* | | | | | |
|  |  | | |  | | |  | | | | *Group: F(2,19)=1.65,p=0.217* | | | | | |
|  |  | | |  | | |  | | | | *Tone Frequency *Group: F(26,713)=2.15,p=000.8* | | | | | |

| **CR peaktime** | | | | | | | | | | **CS- comparison between groups** | | | | | |
| --- | --- | --- | --- | --- | --- | --- | --- | --- | --- | --- | --- | --- | --- | --- | --- |
|  | **Grp.10CS+4CS- ( n = 8 mice)** | | | **Grp.10CS+9CS- (n = 8 mice)** | | | **Grp.10CS+9.5CS- (n =6 mice)** | | | **Grp.10CS+4CS- vs Grp.10CS+9.5CS-** | | **Grp.10CS+4CS- vs Grp.10CS+9CS-** | | **Grp.10CS+9.5CS- vs.GRP.10CS+9CS-** | |
| **CS-only** | **CS-** | **p-value** | **q-value** | **CS-** | **p-value** | **q-value** | **CS-** | **p-value** | **q-value** | **p-value** | **q-value** | **p-value** | **q-value** | **p-value** | **q-value** |
| 2 kHz | 200.80(±20.9) | 1.00 | 1.00 | 305.00(±8.0) | 0.990 | 1.00 | 279.41(±13.5) | 0.976 | 1.00 | 0.896 | 0.979 | 0.604 | 0.979 | 0.305 | 0.979 |
| 4 kHz | **298.48(±12.2)** | **0.993** | **1.00** | 288.97(±15.7) | 0.150 | 1.00 | 308.36(±20.2) | 0.999 | 1.00 | 0.695 | 0.979 | 0.707 | 0.979 | 0.338 | 0.979 |
| 6 kHz | 509.82(±30.2) | 0.993 | 1.00 | 294.34(19.4) | 0.541 | 1.00 | 307.44(±16.0) | 0.998 | 1.00 | 0.923 | 0.979 | 0.673 | 0.979 | 0.448 | 0.979 |
| 8 kHz | 288.92(±11.0) | 1.00 | 1.00 | 312.59(25.5) | 0.997 | 1.00 | 294.28(±9.85) | 0.999 | 1.00 | 0.891 | 0.979 | 0.255 | 0.979 | 0.537 | 0.979 |
| 9 kHz | 312.01(±15.2) | 0.714 | 1.00 | **311.55(8.78)** | **0.999** | **1.00** | 337.74(±16.4) | 0.103 | 1.00 | 0.210 | 0.979 | 0.946 | 0.979 | 0.239 | 0.979 |
| 9.5 kHz | 314.23(±15.6) | 0.265 | 1.00 | 304.23(25.0) | 0.461 | 1.00 | **312.69(±13.6)** | **0.999** | **1.00** | 0.882 | 0.979 | 0.327 | 0.979 | 0.542 | 0.979 |
| ***10 kHz*** | **293.32(±8.19)** | ***1.00*** | **1.00** | **322.40(27.1)** | ***1.00*** | **1.00** | **304.29(±13.9)** | ***1.00*** | **1.00** | ***0.633*** | ***0.979*** | ***0.044*** | ***0.979*** | ***0.320*** | ***0.979*** |
| 10.5 kHz | 298.57(±26.2) | 1.00 | 1.00 | 313.09(27.0) | 0.999 | 1.00 | 304.58(±10.7) | 1.00 | 1.00 | 0.655 | 0.979 | 0.279 | 0.979 | 0.857 | 0.979 |
| 11 kHz | 308.75(±7.55) | 0.593 | 1.00 | 310.41(5.83) | 0.999 | 1.00 | 340.09(±19.9) | 0.111 | 1.00 | 0.232 | 0.979 | 0.949 | 0.979 | 0.335 | 0.979 |
| 12 kHz | 322.28(±13.4) | 0.175 | 1.00 | 313.96(31.9) | 0.908 | 1.00 | 308.76(±11.6) | 0.999 | 1.00 | 0.886 | 0.979 | 0.552 | 0.979 | 0.880 | 0.979 |
| 14 kHz | 306.13(±12.6) | 0.766 | 1.00 | 289.73(16.5) | 0.334 | 1.00 | 309.20(±16.7) | 0.999 | 1.00 | 0.978 | 0.979 | 0.551 | 0.979 | 0.496 | 0.979 |
| 16 kHz | 312.20(±15.2) | 0.131 | 1.00 | 319.69(11.3) | 1.00 | 1.00 | 313.55(±15.7) | 0.992 | 1.00 | 0.979 | 0.979 | 0.890 | 0.979 | 0.814 | 0.979 |
| 18 kHz | 313.39(±12.3) | 0.583 | 1.00 | 309.06(9.04) | 0.999 | 1.00 | 334.68(±24.3) | 0.180 | 1.00 | 0.306 | 0.979 | 0.976 | 0.979 | 0.400 | 0.979 |
| 20 kHz | 304.60(±11.5) | 0.987 | 1.00 | 304.65(20.4) | 0.981 | 1.00 | 325.43(±21.3) | 0.533 | 1.00 | 0.208 | 0.979 | 0.922 | 0.979 | 0.355 | 0.979 |
| *Tone Frequency* | *F(13,1066)=2.12, p=0.011* | | | *F(13,1228)=2.0, p=0.0177* | | | *F(13,839)=3.49,p<.0001* | | | *Tone Frequency:F(13,3133)=3.35,p<.0001* | | | | | |
|  |  | | |  | | |  | | | *Group: F(2,19)=0.39,p=0.681* | | | | | |
|  |  | | |  | | |  | | | *Tone Frequency*Group: F(26,3133)=1.96,p=0.002* | | | | | |

**Supplementary Table 4| Eyeblink conditioning outcome measures during generalization test session (19-23).** All values represent mean ±95%CI. The ANOVA on the linear mixed effect (LME) shows the main effect of sound frequency specifically for some groups. Post-hoc comparisons are shown in Supplementary Table 5. Abbreviation: CR, conditioned response, CS+, reinforced stimulus, CS- non reinforced stimulus.

| **Tone Frequency (kHz)** | **2** | **4** | **6** | **8** | **9** | **9.5** | **10.5** | **11** | **12** | **14** | **16** | **18** | **20** |
| --- | --- | --- | --- | --- | --- | --- | --- | --- | --- | --- | --- | --- | --- |
| Eye closure – all trials (Grp.10CS+4CS-) | <.0001 | <.0001 | <.0001 | <.0001 | <.0001 | 0.189 | 0.393 | 0.001 | 0.006 | 0.355 | 0.026 | 0.279 | 0.013 |
| Eye closure – all trials (Grp.10CS+9CS-) | 0.004 | 0.2872 | 0.016 | 1.14 | 0.025 | 1.56 | 2.13 | 0.521 | 1.42 | 1.74 | 0.706 | 0.777 | 0.729 |
| Eye closure – all trials (Grp.10CS+9.5CS-) | 0.024 | 0.810 | 0.024 | 1.09 | 0.287 | 1.09 | 1.13 | 0.524 | 1.09 | 0.863 | 1.14 | 1.09 | 0.634 |
|  |  |  |  |  |  |  |  |  |  |  |  |  |  |
| Eye closure – CR only trials (Grp.10CS+4CS-) | >.0001 | >.0001 | 0.001 | 0.0008 | 0.002 | 0.221 | 0.4911 | 0.033 | 0.209 | 0.370 | 0.030 | 0.927 | 0.029 |
| Eye closure – CR only trials (Grp.10CS+9CS-) | 0.079 | 1.07 | 1.58 | 1.58 | 0.277 | 1.24 | 2.10 | 0.618 | 1.70 | 1.71 | 1.24 | 1.07 | 1.58 |
| Eye closure – CR only trials (Grp.10CS+9.5CS-) | 0.634 | 0.862 | 0.634 | 1.49 | 0.904 | 1.557 | 1.79 | 1.99 | 2.12 | 1.31 | 2.11 | 1.55 | 0.90 |

**Supplementary Table 5| Post-hoc comparison between CS+ and all the other generalization test frequencies for cumulative amplitude of eyelid closure calculated over all trials and for cumulative amplitude of eyelid closure calculated over CR only trials.** All values represent FDR corrected post-hoc comparisons using a two-sample Kolmogorov Smirnov test on the cumulative distribution. Abbreviation: CR, conditioned response, CS+, reinforced stimulus, CS- non reinforced stimulus.
